# A kinetic model of copper homeostasis in *Saccharomyces cerevisiae*

**DOI:** 10.1016/j.jbc.2025.110368

**Published:** 2025-06-16

**Authors:** Cade Dulaney, Jay R. Walton, Paul A. Lindahl

**Affiliations:** 1 Department of Mathematics, Texas A & M University, College Station, Texas, USA; 2 Department of Chemistry, Texas A & M University, College Station, Texas, USA; 3 Department of Biochemistry and Biophysics, Texas A & M University, College Station, Texas, USA

**Keywords:** mathematical modeling, computational systems biology, simulation, *in silico* cell models, labile metal pools, “free” copper, basic pathways, FET4

## Abstract

Rather than inhibiting copper entry when grown on high Cu, yeast cells import excessive Cu while simultaneously increasing expression of metallothionein CUP1 which then sequesters excess Cu. An ordinary-differential-equations-based kinetic model was developed to investigate this unusual behavior. The assumed reaction network included 25 reactions and 10 components in the cytosol of yeast cells growing in media supplemented with a series of increasing nutrient COPPER concentrations. Published concentrations of copper proteins and coordination complexes that constitutes the low-molecular-mass (or labile) Cu pool were assumed. Other components included transcription factors MAC1 and ACE1, the MAC1-dependent copper importer CTR1, and other copper proteins considered collectively. A second MAC1-independent importer was required for sufficient Cu to enter the cell under Cu-excess conditions. The mathematical system was initially solved at steady state for each condition in the series. The null-space of the stoichiometric matrix was evaluated using the basic pathways approach. Steady-state rates and rate-constants were calculated for each reaction and each condition of the series. Twenty-one rate-constants remained relatively constant across the series, while 4 trended higher, indicating that cells regulate those latter reactions in ways that were not included in their assumed rate-law expressions. This behavior was simulated by augmenting those expressions with logistical functions that sensed labile Cu and/or nutrient COPPER. The resulting integrated dynamical system approximately generated observed component concentrations over the series and was stable to both intracellular and extracellular perturbations. The MAC1-independent importer is predicted to be FET4, a nonspecific importer of both Cu and Fe. Cells may tolerate excessive Cu import to import sufficient iron.

Budding yeast contains less than two dozen copper-containing proteins (Fig. 1, top panel), yet this d-block transition metal is essential for this and all other forms of life (1, 2, 3). CTR1 and CTR3 are specific high-affinity Cu importers on the plasma membrane (4). Mitochondrial intermembrane-space proteins COX17, COX11, SCO1, COX23, and COX19 traffic Cu from the cytosol for final installation into COX1 and COX2, the two Cu-containing subunits of cytochrome c oxidase (5). This enzyme catalyzes the reduction of O_2_ to H_2_O during respiration (6). Chaperone protein CCS delivers Cu to superoxide dismutase SOD1 which detoxifies superoxide, a biproduct of respiration (7). Chaperone protein ATX1 delivers Cu to the Golgi apparatus *via* Cu importer CCC2 for installation into multicopper oxidases FET3 and FET5 (8). FET3 moves to the plasma membrane where it helps import iron, while FET5 is installed in the vacuolar membrane where it helps mobilize stored iron under Fe-deficient conditions. MAC1 and ACE1 are Cu-binding transcription factors that homeostatically regulate cellular Cu (9, 10). Metallothioneins CUP1 and CRS5 sequester excess cellular Cu (11). This is the complete inventory of known Cu proteins in yeast cells. The concentrations of these proteins in such cells have been determined by quantitative mass spectroscopy-based proteomics (Table S1) (12).

**Figure 1:**
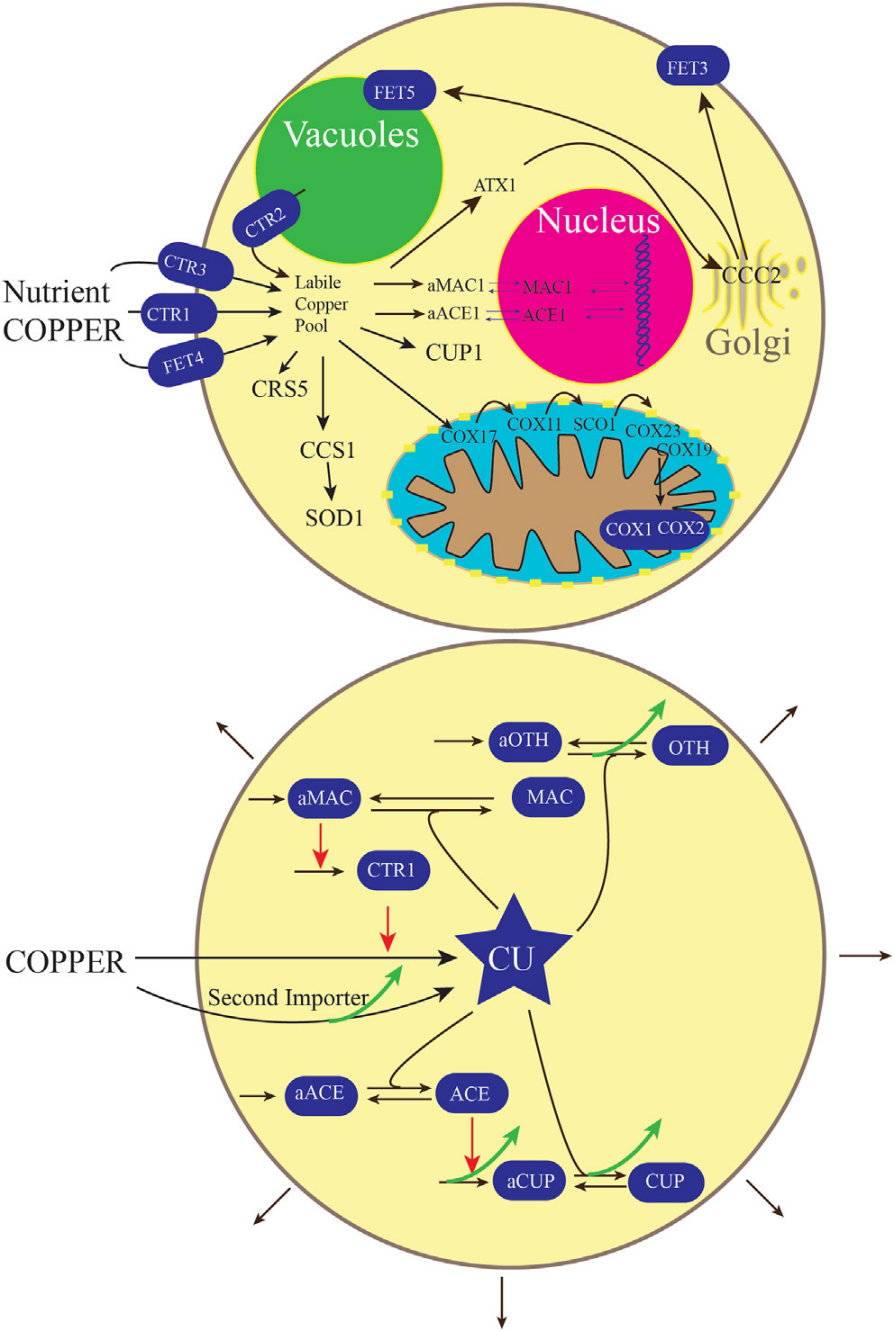
**Copper proteins in yeast cells (top) and modeling outline (bottom).***Toppanel*: overview of copper metabolism in *Saccharomycescerevisiae* cells, as described in the text. *Bottompanel*: reaction network and components used for the *in silico* cell model. The cell grows on nutrient COPPER, with growth indicated by the external outward pointing *arrows*. *Blue shapes* indicate cellular components, *black lines* indicate reactions, *red lines* indicate catalytic influences, and *green lines* indicate a stimulation of the indicated reaction as [COPPER] increases.

Our objective was to develop an ordinary-differential-equation (ODE)-based mathematical model of copper homeostasis within these cells, highlighting the role of the labile copper pool (CU) as indicated by the blue star in the bottom panel of Figure 1. No such models have been attempted previously because insufficient kinetic information was available. This includes unknown rate-constants *k*_*rxn*_, Michaelis–Menten *K*_*m*_ constants, and protein concentrations. However, we were able to use the recent results of Kim *et al.* (13) along with other published data to generate the model described here. Kim *et al.* grew batches of yeast cells in minimal respiring media at seven different nutrient CuSO_4_ concentrations. One batch of this so-called *M* titration, called *MBCS*, lacked CuSO_4_ and contained the Cu chelator bathocuproine sulfonate. Other batches, called *M0*, *M10*, *M50*, *M100*, *M175*, and *M250* lacked the chelator and were supplemented with the indicated concentration of CuSO_4_ in μM. Cytosol was isolated from each batch and analyzed by liquid chromatography with inline inductively-coupled-plasma-mass-spectrometry (ICP-MS) detection of Cu. Approximately 27 partially resolved Cu-bound species were detected and quantified, including about a dozen Cu-bound proteins and a similar number of low-molecular-mass (LMM) Cu complexes. Peaks arising from CUP1 and SOD1 were identified, but the other Cu proteins were not. For simplicity, the resulting Cu peaks were organized into groups, summarized in Table S2 and illustrated in Figure 2, top panel. CUP1 and adjacent partially resolved minority peaks will be called the **CUP** group (dark purple). MAC and ACE are shown in green. SOD1 and a half-dozen other proteins (other than those assigned to CUP) will be called the **OTH** group (blue), and the dozen or so LMM Cu complexes will be collectively defined as the labile Cu pool **CU** (red).

**Figure 2:**
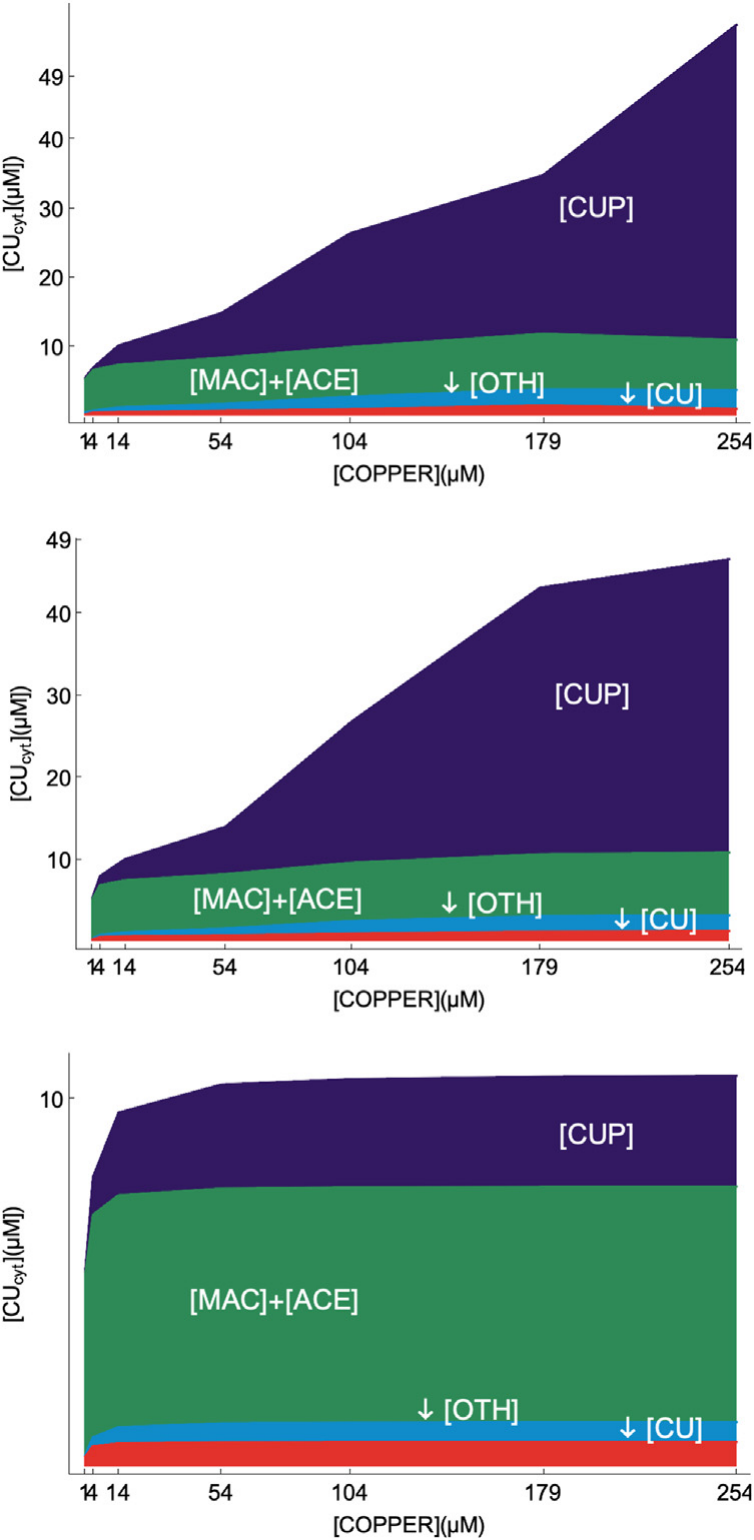
**Copper concentration of the cytosol isolated from cells grown on increasing nutrient COPPER concentrations.***Toppanel*: Cu content of yeast cytosol including all soluble Cu-bound species reported similar to Figure 5 in Kim *et al.* (13). *Dark blue* indicates CUP1, *green* indicates MAC1 and ACE1, *light blue* indicates OTH (other) proteins, and *red* indicated CU. *Middlepanel*: same, but with concentrations predicted by WT model simulation. *Bottompanel*: same but with concentrations predicted by the “mutant” model in which the second importer has been deleted.

Results from quantitative mass spectrometry proteomics studies (12) were also used in developing the model. However, the reported protein concentrations were generally obtained using cells that had been grown (fermented) on glucose-rich media; such media have significant but unmeasured concentrations of nutrient copper. In contrast, cells used by Kim were grown on nonfermenting minimal media supplemented with increasing concentrations of CuSO_4_. Moreover, Kim measured *copper* concentrations whereas proteomics studies measured *protein* concentrations. Converting from one to the other requires knowing the number of coppers bound per protein (Cu stoichiometry) as well as the fraction of each protein that is Cu-bound (fractional occupancy). To mitigate these uncertainties, we assumed that the cells of the M10 batch of Kim (13) were identical to those analyzed in the proteomic studies. This approximation was required for model development. The soluble cytosolic fraction of M10 contained 4.0 μM Cu, 3.4 μM of which was bound to proteins and 0.6 μM bound to LMM complexes. Kim estimated the fractional occupancy to be ∼ 0.30 under these conditions (Table S2). Assuming the same fractional occupancy for the proteomics calculations (Table S1) suggests that the soluble fraction of cytosolic proteins should contain 2.1 μM Cu, sufficiently near to Kim’s value of 3.4 μM to conclude that our assumptions were reasonable.

### The labile copper pool

Establishing that cells contain such pools, defined here as nonproteinaceous LMM Cu coordination complexes located in aqueous regions of the cell, has had a contentious 36-years history. The concept was introduced in 1989 when Peisach and coworkers reported that > 60% of the Cu in the cytosol of hepatoma cells (grown in media containing 600 μM Cu) was composed of Cu coordinated to glutathione (Cu-GSH) (14), a thiol-containing metabolite present in high (mM) cellular concentrations. A few years later, the Kosman group used liquid chromatography to investigate the copper content of cytosolic extracts of *Saccharomyces cerevisiae.* They reported a smaller pool of LMM Cu that also comigrated with Cu-GSH (15, 16). Both conclusions were challenged in 1999 when Rae *et al.* discovered that apo-SOD1 from *S. cerevisiae* could not be metallated in cells lacking the SOD1-chaperone CCS (17). Since Cu-GSH *could* metallate apo-SOD1 *in vitro*, Rae *et al.* reasonably concluded that cells must not contain this Cu coordination complex, and they reinforced this conclusion with calculations showing that the concentration of “free” copper in cells must be < 10^−18^ M. They argued that the absence of a labile pool in cells was due to the presence of metallothioneins CUP1 and CRS5 which scavenged any and all “free” Cu. Although the term “free” was left undefined, their usage implied inclusion of both aqueous *Cu* (copper coordinated exclusively by rapidly exchanging waters and or hydroxide ions) and Cu-GSH.

Soon thereafter, Wedd and coworkers titrated a number of Cu-binding proteins with aqueous Cu and obtained thermodynamic Cu-dissociation constants of order 10^−19^ M (18), supporting the results and conclusions of Rae *et al.* Wegner *et al.* performed similar titrations of MAC1 and ACE1, and obtained *K*_*d*_ values in the same ballpark (9.7 × 10^−20^ M and 4.7 × 10^−18^ M, respectively) (10). These constants were viewed as representing the intracellular “free” Cu^I^ concentration at 50% activation of the titrated proteins, assuming the generic dissociation reaction Equation 1.

(1) $$Cu\cdot P\overset{K_{dPa}}{⇄}\;Cu\left(aq\right)+P$$

In this equation, *Cu(aq)* refers to aqueous Cu, *P* to a generic apo-protein, and *Cu·P* to the Cu-bound form of P. Subscript *a* in *K*_*dPa*_ indicates that aqueous Cu was used in the titration or, more precisely, that this *K*_*d*_ would have been obtained had aqueous Cu been used. In reality, *K*_*dPa*_ was so small that titrations had to be performed using Cu coordinated to a strong-binding chelator such as bathocuproine disulfonate or cyanide. Thus, the dissociation reaction associated with the actual titration is more accurately described by Equation 2

(2) $$Cu\cdot P+L\overset{K_{dPL}}{⇄}Cu\cdot L+P$$

where L is the chelator used. Subscript L in *K*_*dL*_ refers to the use of a copper complex in the titration. *Cu·L* complexes were selected such that *K*_*dLa*_ for the chelator complex, associated with reaction Equation 3

(3) $$Cu\cdot L\overset{K_{dLa}}{⇄}\;Cu\left(aq\right)+L$$

was known. This allowed *K*_*dPa*_ to be calculated as the product of measured *K*_*dPL*_ and the previously determined *K*_*dLa*_. For example, if *K*_*dLa*_ had been reported to be 10^−16^ M and *K*_*dPL*_ was measured to be 10^−2^ (unitless), then *K*_*dPa*_ would equal 10^−18^ M. Then, according to Equation 1, *Cu(aq)* would equal that *K*_*dPa*_ when the ratio of apo-to-holo forms of the protein, [P]/[Cu·P] = 1. However, given the volume of a yeast cell, a *Cu(aq)* concentration of 10^−18^ M corresponds to about 1 Cu atom per 40 million cells, indicating that for all practical purposes, cells are devoid of aqueous copper. We conclude that *aqueous Cu must not be involved in copper homeostasis*.

Meanwhile, studies in which intact cells were exposed to custom-designed fluorescence-based chelator probes or sensors of *Cu(aq) and* probably of labile Cu complexes detected some such Cu species in cells. In 2005, Yang *et al.* detected a labile pool in mouse fibroblasts using a membrane-permeable Cu^I^-binding fluorescence sensor (19). A few years later, Dodani *et al.* reported a Cu pool in HEK cells also using a fluorescence Cu-binding sensor (20). Numerous subsequent studies also detected labile Cu pools (21).

Most of these results can be reconciled with those of Kim *et al.* (13) by assuming that the chelator probes were detecting labile Cu pools, not aqueous Cu. In this case the Cu trafficking reactions occurring in a cell would corresponding to the reverse of Equation 2, where *K*_*dPL*_, *K*_*dPa*_, and *K*_*dLa*_ would equal something like 10^−2^, 10^−18^, and 10^−16^ M, respectively (presuming that P binds aqueous Cu 100 × more tightly than does L). If [L] would equal 10^−4^ M, a typical concentration for a potential Cu-binding metabolite in the cytosol, the apparent dissociation constant *K*_*dPL*_*'* = *K*_*dPL*_·[L] = 10^−2^·10^−4^ M = 10^−6^ M. Then, at a 1:1 M ratio of [P]/[Cu·P] the concentration of *CU* would be 1 μM, the same order of magnitude as detected by Kim. In that case, a yeast cell of volume 42 × 10^−15^ L would then contain about 25,000 atoms of labile Cu, a concentration detectable by fluorescent probes.

But how can this scenario be reconciled with the conclusion of Rae *et al.* that cells are not only devoid of *Cu(aq)* but also of *Cu-GSH*? The activation properties of apo-SOD1 were later found to be more complicated than realized at the time. Culotta and coworkers discovered that activating apo-SOD requires not merely installing Cu but also oxidizing two cysteines to form a disulfide bond (22). The inability of yeast apo SOD to be activated by Rae *et al.* in ΔCCS1 cells may not have been due to insufficient Cu-GSH in the cytosol, but to the reducing cellular conditions that prevented these cysteines from being oxidized. Indeed, Leitch *et al.* concluded that CCS and apo-SOD compete for a common pool of intracellular copper, which they proposed to be Cu-GSH (22).

### Homeostatic regulation of the low mass Cu pool

Total cellular Cu in general and CU in particular are thought to be homeostatically regulated by a mechanism involving MAC1, ACE1, CUP1, and CTR1. These interactions constitute the foundation of our model (Fig. 1, bottom). Under Cu-deficient conditions, the majority of MAC1 is present in its apo form (**aMAC** in our model—all model components are introduced in bold), and this form stimulates expression of **CTR** which imports nutrient **COPPER**. Under Cu-excess conditions, apo-ACE1 (**aACE**) becomes metallated, and the resulting form **ACE** stimulates expression of **aCUP**. Resulting aCUP sequesters excess cytosolic CU. Wegner *et al.* (10) described the two opposing processes as creating a “window” within which cells regulate the concentration of “free” cytosolic Cu. They plotted the percent deactivation of aMAC and percent activation of aACE as a function of reduced “free” Cu, reproduced in Figure 3, top panel.

**Figure 3:**
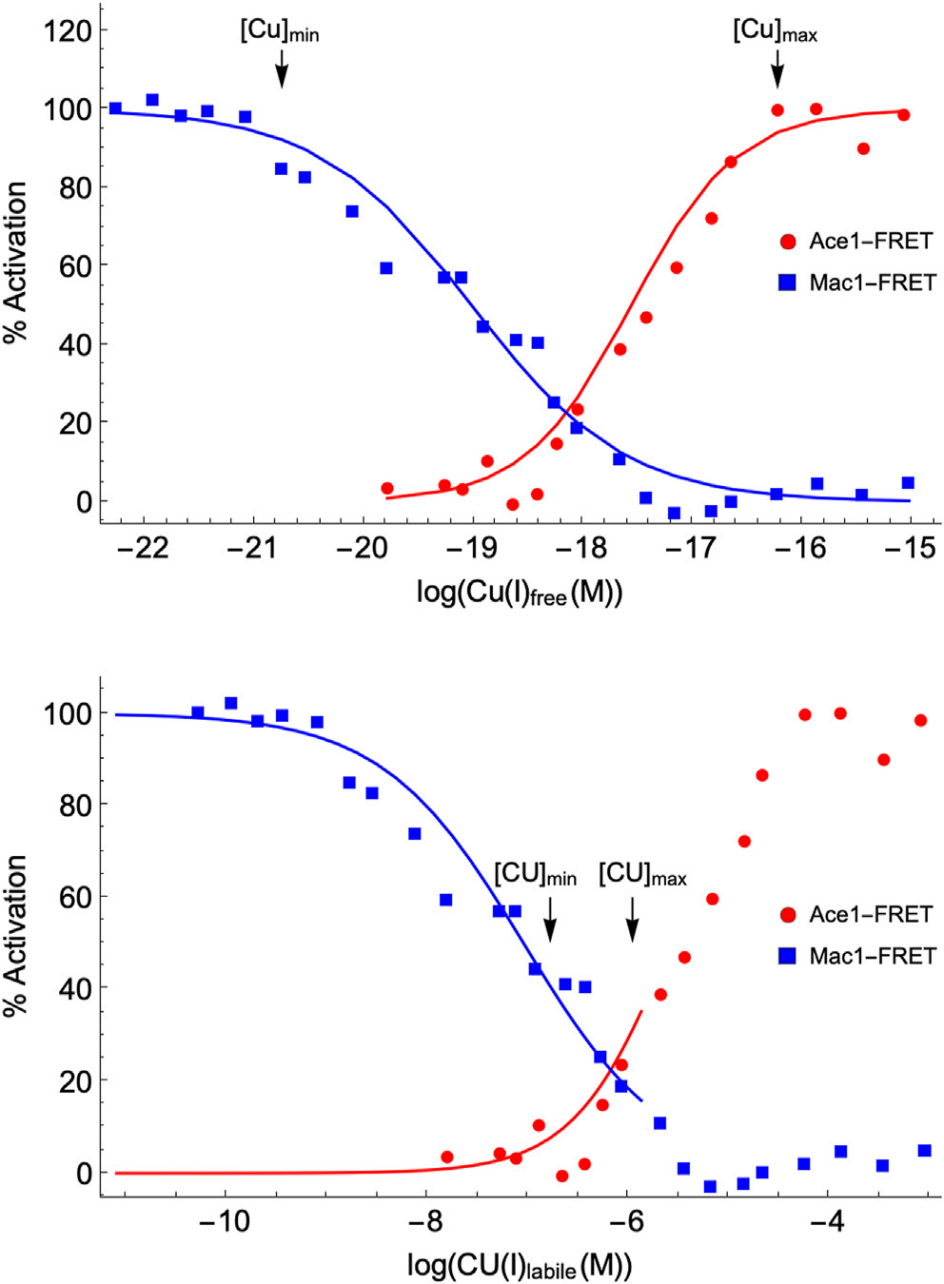
**Plot of MAC deactivation and ACE activation with increasing concentrations of “free” Cu (*top*) and the labile Cu pool CU (*bottom*).***Toppanel*: Data points were manually estimated from the original plot in Wegner *et al.* (10). *Blue* and *red* simulation lines were obtained using Equations 5 and 6, as described in the text. Min/max *arrows* were also taken from Wegner (10). *Bottompanel*: Data points are the same as above but concentrations on the abscissa were multiplied by 9.67 × 10^11^ to convert the “free” Cu concentrations into the concentration of the labile Cu pool (CU). Min/max *arrows* were from concentrations of CU obtained by Kim *et al.* for Cu-deficient and Cu-excess conditions (13).

Both MAC1 and ACE1 are housed in the nucleus (23, 24), and both contain cysteine-rich domains which bind 4 Cu ions to form tetracopper clusters (25). Whether these ions bind cooperatively is plausible but unestablished. Wegner *et al.* observed the binding of four equivalents of Cu to aACE and aMAC (refer to their Fig. 3, *A* and *B* (10)). To consider this possibility, we resimulated the Wegner titration data by assuming Equation 4

(4) $$Cu\cdot P\overset{K_{dMAC}\;or\;K_{dACE}}{⇄}nCU+P$$

where *n* is the Hill coefficient associated with the metallation of P. Substituting [P_tot_] = [P] + [Cu·P] into the expression describing K_d_ in Equation 4 yields Equation 5 after rearrangement.

(5) $$\frac{1}{\left(\frac{{\left[CU\right]}^{n}}{K_{d}}+1\right)}=\frac{\left[P\right]}{\left[P_{tot}\right]}$$

Note that when [P]/[P_tot_] = 0.50, $K_{d}={\left({\left[CU\right]}_{0.5}\right)}^{n}$. In words, *K*_*d*_ equals [CU]_0.5_ raised to the *n*^th^ power. Substituting this into Equation 5 and rearranging yielded Equation 6.

(6) $$n=\frac{\log\left(\frac{\left[P_{tot}\right]}{\left[P\right]}-1\right)}{\log\left(\frac{\left[CU\right]}{{\left[CU\right]}_{0.5}}\right)}$$

For MAC, [CU]_0.5_ ≈ 9.7 × 10^−20^ M. Using this value, Equation 6 was solved using the middle 14 points of the MAC data in the Wegner plot. This afforded average *n* ≈ 0.62 and thus *K*_*dMAC*_ ≈ 1.6 × 10^−12^ M. Other informative conditions are when [P]/[P_tot_] = 0.90 and 0.10. Solving Equation 5 under these conditions reveals that [CU]_0.1_/[CU]_0.9_ = $\sqrt[{n}]{81}$. Reading off the plot of Figure 3, bottom panel, [CU]_0.1_/[CU]_0.9_ ≈ 1200 (3.1 μM/2.8 nM) which again indicates *n* ≈ 0.62. If *n* equaled 4, the same ratio would have equaled 3. Similar analysis for ACE yielded [CU]_0.5_ ≈ 2.8 × 10^−18^ M, *K*_*dACE*_ ≈ 1.6 × 10^−16^ M, and *n* ≈ 0.90. (For CUP, *K*_*dCUP*_ ≈ 9.3 × 10^−19^ M and *n* ≈ 1.1.) Thus, the possibility of positive cooperative binding can be excluded by visual inspection of the Wegner plot. Positive cooperativity, indicated by Hill coefficients *n* > 1, would occur if binding the first Cu to a protein caused subsequent Cu ions to bind with greater affinity. We expected this for aMAC and aACE because each contains cysteine-rich regions that can accommodate tetracopper clusters. Negative cooperativity, with *n* < 1, would occur if binding the first Cu caused subsequent Cu ions to bind with lower affinity. Noncooperativity (*n* = 1) would occur if the binding strength of each Cu ion were equal and unaffected by the others. Thus, *n* does not necessarily indicate the number of Cu ions that bind per protein, an unexpected condition that must be assumed here. The Wegner plot of Figure 3 indicates negative cooperativity for MAC and within error of noncooperativity for ACE and CUP. These conclusions are admittedly nonintuitive.

## Results

### Recalibrating the Wegner plot

We wanted to utilize the Wegner data in developing our model, but after concluding that “free” (*i.e.* aqueous) Cu is irrelevant for copper homeostasis, we realized that their plot needed to be recalibrated in terms of the labile Cu pool concentration. This was done by assuming that the [Cu^I^]_free_ concentration at the average of pK_dMAC_ and pK_dACE_ (6.1 × 10^−19^ M) was equal to the labile Cu pool concentration in *M10* cytosol, namely [CU] = 0.59 μM. We then multiplied the original [Cu^I^]_free_ values by the unitless recalibration factor [CU]_M10_/[Cu^I^]_free_, namely 9.7 × 10^11^, to generate Figure 3, bottom panel. Doing this had no effect on the Hill coefficients obtained using the original Wegner plots.

### Estimating protein concentrations at different [COPPER]

Solving the model at steady-state required that steady-state concentrations of all model components for each of the seven conditions MBCS, …M250 be assigned. To do this, we estimated the steady-state concentrations of the three Cu-containing species detected by Kim *et al.*, namely CUP, OTH, and CU (Table S2), for each of the 7 *M* titration conditions (13). However, Kim reported copper concentrations which were fine for determining the CU concentration. However, obtaining protein concentrations, as needed for the model, required solving Equation 7 (using CUP to illustrate).

(7) $${\left[CUP\right]}_{tot}={\left[Cu\right]}_{CUP}\cdot \frac{{\left[CUP\right]}_{holo}}{{\left[Cu\right]}_{CUP}}\cdot \frac{{\left[CUP\right]}_{tot}}{{\left[CUP\right]}_{holo}}$$

In words, Equation 7 indicates that the concentration of CUP protein equals the Cu concentration associated with CUP (as detected by the chromatograms) divided by the number of coppers bound per CUP and divided by the fraction of CUP proteins bound with copper. For *M10*, [Cu]_CUP_ was 2.8 μM. With eight copper ions bound per CUP, and an assumed fractional occupancy of 0.30, [CUP]_tot_, [CUP]_holo_, and [CUP]_apo_ were calculated to be 1.2, 0.35, and 0.80 μM, respectively. For OTH, [Cu]_OTH_ was 0.67 μM and 1 Cu bound per OTH protein. Assuming the same fractional occupancy yielded [OTH]_tot_, [OTH]_holo_, and [OTH]_apo_ of 2.2, 0.67, and 1.6 μM, respectively. For comparison, [CUP]_tot_ and [OTH]_tot_ obtained by proteomics were 0.24 and 5.2, respectively. These differences in protein concentration estimates are considerable for [CUP]_tot_ (1.2 versus 0.24 μM). We suspect that CUP is more highly expressed under Kim's M10 conditions than in the conditions used in the proteomic studies (rich media without Cu supplementation). The expression levels of the other Cu proteins may have changed less with growth conditions, given the smaller difference (2.2 versus 5.2 μM).

Estimating the concentrations of MAC and ACE for all 7 M titration conditions required a different approach, because these proteins are in the nucleus rather than cytosol, so they were not measured by Kim. Proteomics data for these two proteins were only available under one condition, and so we assumed that the total concentrations of these two proteins remained invariant for all 7 M conditions. We relied on the recalibrated Wegner plot (Fig. 3, bottom panel) to estimate the ratios of apo and holo states for each condition, affording the results in Table S2.

Estimating the concentrations of CTR for all 7 M conditions was also a challenge since only one proteomics cellular concentration was available, which we assumed reflected *M10* conditions. CTR is membrane bound, but the model assumed a homogenous cytosol; this allowed a local concentration of 0.29 μM to be calculated. Since aMAC promotes the expression of *ctr1/3* genes, the [CTR] for each M condition was presumed to be proportional to [aMAC] under the same condition. This allowed us to estimate the CTR concentrations for the remaining six titration conditions, by multiplying [aMAC] at each condition by 0.79, the ratio of [CTR]_M10_/[aMAC]_M10_ (0.29/0.37). Again, results are listed in Table S2.

### Unusual homeostatic behavior

Given the cellular need for Cu and the toxicity of Cu to cells, our expectation for the M titration of Kim was that cells would limit Cu import once they contained sufficient Cu to support healthy growth (*i.e.* M10). In contrast, Kim found that cells imported excessive Cu unabated at high nutrient copper concentrations while simultaneously increasing expression of aCUP which then sequestered most of the excess Cu (Fig. 2, upper panel).

Distinguishing species that are *regulated* from those that are *regulating* can help explain such unexpected behavior. In the developed model, components CU, aOTH, and OTH are regulated whereas aMAC, MAC, aACE, ACE, aCUP, CUP, and CTR are regulating. In effective homeostatic mechanisms, the concentrations of the regulated groups should change minimally as [COPPER] changes, whereas the concentration of components in the regulating group, or at least their ratios of apo versus holo forms, might change majorly. Homeostasis of CU and OTH should be most effective at intermediate levels of [COPPER], namely under the M10 condition, and less so under Cu-starved (MBCS) and Cu-excess (M250) conditions. Meanwhile, the concentrations of the components of the regulating group, especially CUP, can and do change dramatically.

Still unanswered is why cells utilize this form of homeostasis, rather than simply restricting Cu import under Cu-excess conditions. Indeed, addressing this puzzle motivated us to develop this model of copper homeostasis. Such models are perhaps uniquely qualified to integrate the various disparate factors that maintain cellular homeostasis in a changing environment, and perhaps highlight deficiencies in current understandings.

### The biochemical reaction network

The biochemical reaction network that defined the model, illustrated in Figure 1, bottom panel, was composed of 25 reactions (Table S3) and 10 components, all contained in the cytosol of a growing yeast cell. One limitation of the model is that it does not include vacuoles, acidic organelles in yeast that store excess Cu; insufficient quantitative information was available to justify their inclusion.

The same reaction network was assumed for all 7 *M* titration conditions. Five reactions represented the biosynthesis of proteins aMAC, aACE, aCUP, aOTH, and CTR. For example, reaction *BAMAC*, Equation 8,

(8) →aMAC

represented the biosynthesis of aMAC. The same name doubled for the rate of the reaction. These reactions lack substrates (aMAC seems to be created from nothing) because the substrates needed to synthesize proteins were not our focus. Excluding them is tantamount to assuming that their concentrations remain constant under all modeling conditions.

*BAMAC* and two other biosynthesis reactions (*BAACE* and *BAOTH*) were uncatalyzed while *BCTR* and *BACUP*, were catalyzed by aMAC and ACE, respectively. Two reactions, *CUIN1* and *CUIN2* describe the import of nutrient COPPER forming *CU* as the product, as in Equation 9.

(9) COPPER→CU

*CUIN1* was catalyzed by CTR whereas *CUIN2* was regulated by the logistical function described below. Including the second importer was an unexpected requirement for modeling observed behavior.

Each protein in the model except CTR had two forms, apo and holo. Each apo form could be reversibly metallated as shown for aACE in Equation 10.

(10) $$aACE+4CU\overset{MACEF}{\underset{MACER}{⇌}}ACE$$

*MACEF* and *MACER* are the forward and reverse reactions, respectively. aMAC, aACE, and aCUP were presumed to bind 4, 4, and 8 coppers, respectively. These stochiometric *n* values were included in the stochiometric matrix, as they indicate how many Cu ions bind per protein, not whether the binding is cooperative. Different values of *n* were used in the corresponding rate law expressions (see below); these *n* values reflect the extent of cooperative binding.

Each component of the model was presumed to be located in growing *in silico* yeast cells. Increasing cell volumes cause component concentrations to decline, and this was represented as dilution reactions. For example, dilution of OTH (*DOTH)* was given by Equation 11.

(11) OTH→

There are no products in these reactions because they simply reflect dilution. All ten components were diluted in this manner.

### Rate law expressions

The rate of each reaction was presumed to be influenced by the concentrations of substrates and catalysts according to the rate law expressions in Table S3. These expressions represent major assumptions of the model since none has been determined experimentally. The form assumed for these expressions depended on whether the reaction was presumed to be enzyme catalyzed. If so, a Michaelis–Menten-like equation was assumed, and if not, mass-action kinetics for each substrate were assumed. *CUIN1* was catalyzed by CTR, and so its rate law expression was

(12) $$CUIN1=kCUIN1\cdot \left(\frac{\left[COPPER\right]}{K_{m\left(1\right)}+\left[COPPER\right]}\right)\left(\frac{\left[CTR\right]}{{\left[CTR\right]}_{M10}}\right)$$

Hassett et al., determined *K*_*m*__(__*1*__)_ for CTR of 2 μM (26) and this value was assumed. The catalytic influence of CTR was normalized by dividing its concentration by the steady-state *M10* concentration given in Table S2. The expression for *CUIN2* was similar except it was not catalyzed by CTR and *K*_*m(2)*_ was 35 μM, as determined for the nonspecific importer FET4 (27). The rate law expression for CUIN2 was further modified with a logistical function as described below.

Metallation and demetallation reactions were presumed to be uncatalyzed. The rate law expressions for the metallation of aMAC and demetallation of MAC are given by Equation 13.

(13) $$\begin{array}{l} MMACF=kMMACF\left(\frac{\left[aMAC\right]}{{\left[aMAC\right]}_{M10}}\right){\left(\frac{\left[CU\right]}{{\left[CU\right]}_{M10}}\right)}^{0.62}\\ MMACR=kMMACR\left(\frac{\left[MAC\right]}{{\left[MAC\right]}_{M10}}\right) \end{array}$$

ACE, CUP, and OTH had similar forms. Rate law expressions for metallation reactions assumed the cooperativity *n* values derived from the Wegner plot (*i.e. n* = 0.62, 0.90, and 1.1) as described above.

### The stoichiometric (S) matrix

The 10 ODEs in the system describe the concentration-change of each component. Each was composed of the sum or difference of the rates in which the component was involved, multiplied by a stochiometric coefficient. For example, the ODE describing how the concentration of aMAC varies is given by Equation 14.

(14) $$\frac{d\left[aMAC\right]}{dt}=1\cdot BMAC-1\cdot MMACF+1\cdot MMACR-1\cdot DAMAC$$

Stochiometric coefficients were ±1 in this example because 1 mol of aMAC was involved in each reaction; however, this was not always the case. The set of 10 stoichiometrically balanced ODEs were organized into matrix form

(15) $$\left[\begin{array}{ccc} s_{1,1} & ... & s_{1,25}\\ | & | & |\\ s_{10,1} & ... & s_{10,25} \end{array}\right]\left[\begin{array}{l} R_{1}\\ |\\ R_{25} \end{array}\right]=\left[\begin{array}{l} \frac{dC_{1}}{dt}\\ |\\ \frac{dC_{10}}{dt} \end{array}\right]$$

where the left most matrix of 10 rows, each representing a component, and 25 columns, each representing a reaction rate, is the stochiometric or ***S*** matrix (see Table S4). This is followed by ***R*_*cell*_**, a vector of 25 reaction rates, and [dC/dt], a vector of 10 time-dependent component concentration derivatives. At steady-state, [d[C]/dt] becomes the zero vector, allowing the null space of ***S*** to be analyzed using the recently developed basic pathways approach (28). Accordingly, the ***S*** matrix was transformed into a (25 × 15) ***G*** matrix whose columns represent a fundamental algebraic null basis of ***S***. Using a Gauss elimination-inspired algorithm, ***G*** was transformed into another matrix of the same dimension, called the ***W*** matrix, in which all entries are nonnegative. Thus, each column of ***W*** (Table S5) represents a physically meaningful basic pathway. Such pathways include a subset of all the reactions that work together in the network—as dictated by the stoichiometry of the participating reactions—to generate 15 “stoichiometric flows” through the system. Basic pathways typically (but not necessarily) start with nutrients and terminate at a particular model component. A complete list of these pathways reaction network is illustrated in Figure S1.

### Calculating steady-state rates

The next objective was to calculate seven sets of 25 rates, one for each of the 25 reactions of the network operating in each of the 7 M titration conditions. This was problematic as none of these rates had been measured. However, the basic pathways approach provided considerable constraints. Once the ***W*** matrix was generated, the steady-state rates of each reaction in ***R*_*cell*_** obeys the relationship ***R*_*cell*_** = ***W***·***C*_*BP*_** where ***C*_*BP*_** is an n = 15 vector of unknown coefficients c_1_…c_15_, one for each basic pathway. The inherent sparsity of the ***W*** matrix (Table S5) was helpful in solving these relationships. Fifteen rates were independent of the others and had to be assigned values, whereas the remaining 10 were dependent and could be calculated once all independent rates were assigned. The basic pathways approach provides flexibility in these assignments, allowing us to assign the rates of all 10 dilution reactions, one for each component, as being independent. Each dilution rate equaled the exponential growth rate of cell cultures (α_cell_ = 0.0033 min^−1^) multiplied by the steady-state concentration of that component (Table S2). Five other reactions, namely *MMACR*, *MACER*, *MCUPR*, *MOTHR*, and *CUIN2* were also assigned to be independent. Values for the first four reactions had to be guessed because no experimental values were available. We reasoned that metallation and demetallation reactions should be faster than the rate of cell growth, and that these highly reversible reactions should nearly be at equilibrium when the cell is growing at steady-state. We ultimately assigned the rates of demetallation to be 100 × faster than the corresponding rates of dilution. Actual demetallation rates might be faster but assuming this would have increased the stiffness of the system.

Assigning the rate CUIN2 required a different strategy. All nutrient COPPER must enter the cell *via* the two importers, but the fraction entering through each was unknown. To estimate CUIN2, we assumed that all Cu that entered cells grown under Cu-deficient (MBCS) conditions (5.5 μM) originated from CUIN1, and that this process occurred at a rate of {5.5 μM · 0.0033 min^−1^} = 0.018 μM/min. Then, using the rate law expression for CUIN1, we calculated the corresponding rate-constant. Using that rate-constant, the rate of CUIN1 at each of the other six conditions was calculated according to the rate-law expression for CUIN1 and the steady-state concentration of CTR at each condition. CUIN2 was calculated to be the difference between the total rate of Cu entering the cell and the rate due to CUIN1. Once all independent rates were assigned, the dependent rates were calculated immediately. See Table S6 for the complete list of steady-state reaction rates.

### Determining rate-constants

With steady-state rates assigned for each reaction operating under each of the seven titration conditions, the next step was to calculate corresponding rate-constants. Although rates should change as dictated by their rate-law expressions, rate-constants should be invariant for any and all component and nutrient concentrations. To investigate whether this requirement was realized, apparent rate-constants were calculated by substituting the steady-state rates from Table S6 and the steady-state concentrations from Table S2 (or [COPPER] for each condition) into the corresponding rate-law expressions. Twenty-one out of 25 rate-constants were essentially invariant across the titration series (Table S7), as anticipated, whereas rate-constants for *BACUP*, *MCUPF, MOTHF*, and *CUIN2* trended higher (Fig. 1, bottom panel, green arrows). This unexpected result was interpreted as evidence that the cell regulates those four reactions in ways that were not explicated in the assumed rate-law expression. To rectify this, we augmented those expressions with a soft Heavyside logistical function, Equation 16, similar to what we did for an iron trafficking model (29).

(16) $$k_{apparent}=\frac{k_{\text{invariant}}}{1+e^{n\left(Sen_{sp}-Sen\right)}}$$

In Equation 16, *n* (no connection to cooperativity *n*) is a sensitivity factor, *Sen* is the concentration of the sensed species, and the *Sen*_*sp*_ is the setpoint concentration of *Sen* at which *k*_*apparent*_ is midway between 0 and its maximum value *k*_*invariant*_. For the first three of these reactions, *Sen* was CU, while for reaction CUIN2, presumed to be associated with an importer on the plasma membrane, *Sen* was COPPER. Augmenting the rate-law expressions for these four reactions with this function was tantamount to assuming that the cell, through an unknown feedback mechanism, senses CU and/or COPPER, and adjusts the expression levels of the genes/proteins that control the rates of these reactions. Values for *k*_*invariant*_, *Sen*_*sp*_, and *n* were obtained by nonlinear regression using DESMOS (https://www.desmos.com). The datasets used were apparent *k*_*MOTHF*_, *k*_*MCUPF*_, *k*_*BACUP*_, and *k*_*CUIN2*_, as listed in Table S7, versus [CU] (or [COPPER]), as listed in Table S2. Resulting parameters are given in Table S8. Due to the limited data available, invariant *k*_*MOTHF*_, *k*_*MCUPF*_, and *k*_*BACUP*_ values had to be manually constrained within an acceptable range whereas optimized values for *Sen*_*sp*_ and *n* were obtained without manual constraints.

### Engaging the dynamical system

At this point, all of the parameters required to solve the full ODE system (including rate-law expressions) were assigned, and so the system could be and was integrated using steady-state concentrations for each of the seven titration conditions as initial concentrations (Table S2). The resulting dynamical system was attracted to a stable steady-state at all nutrients [COPPER]. Each such state afforded a slightly different set of component concentrations than those used initially (also listed in Table S2). This annealing process occurred because most of the rate-constants used (Table S8) were the average of the slightly different values obtained for each of the seven separately solved conditions as given in Table S7. Moreover, the optimized values for the logistical functions did not precisely generate the apparent rate-constants determined for each condition. The annealing process essentially self-corrected for many minor parameter discrepancies made in constructing the dynamical system.

### Perturbing a component concentration

**Figure 4:**
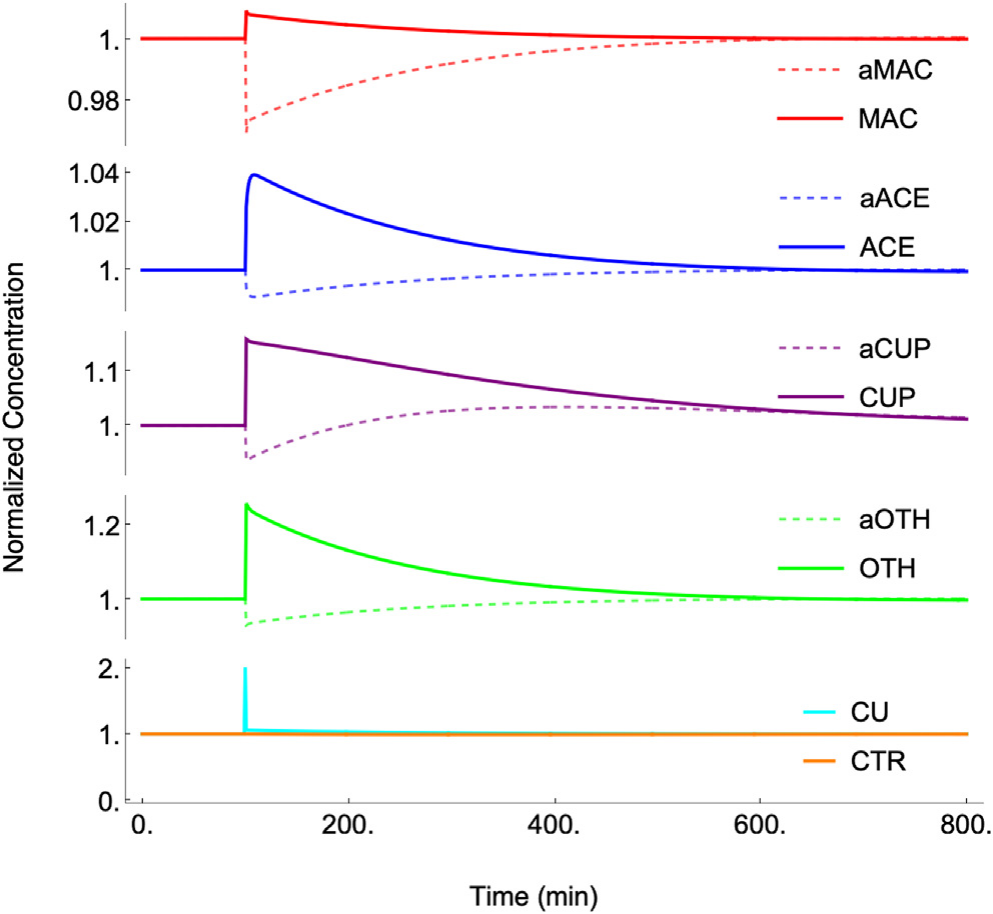
**Recovery of the dynamical system after doubling the concentration of CU at *t* = 100 min with [COPPER] fixed at 14 μM**.

The behavior of the annealed dynamical system was explored by perturbing it and observing the response. Most importantly, the system responded to a perturbation in any component concentration by returning to the stable steady-state. We regard this as required behavior for any model of homeostatic regulation. Figure 4 shows this recovery after abruptly doubling the concentration of CU once the *in silico* cell had been growing for 100 min. Full recovery required ∼ 600 min, about three cell doublings. The average recovery half-life of the non-CU components was between 100 and 250 min whereas that for CU was far faster. These differences arose from differences in the assigned rates of reactions. The rates of metallation/demetallation reactions, all of which involved CU, were about 40-times faster on average than the other rates in the system. Recovery rates of non-CU reactions were largely controlled by the growth rate of the cell, as dilution was the only decay/decomposition-like processes included in the network. Knowledge of experimental recovery rates of intracellular components would constrain reaction rates further.

A related measure of homeostatic effectiveness is the size of a perturbation that the system can withstand yet return to its original state of attraction. The current system recovered from perturbations at least 200 times the nominal concentration of CU, likely higher. This helps quantify the robustness of the modeled regulatory mechanism.

### Dynamic effects of changing nutrient [COPPER]

**Figure 5:**
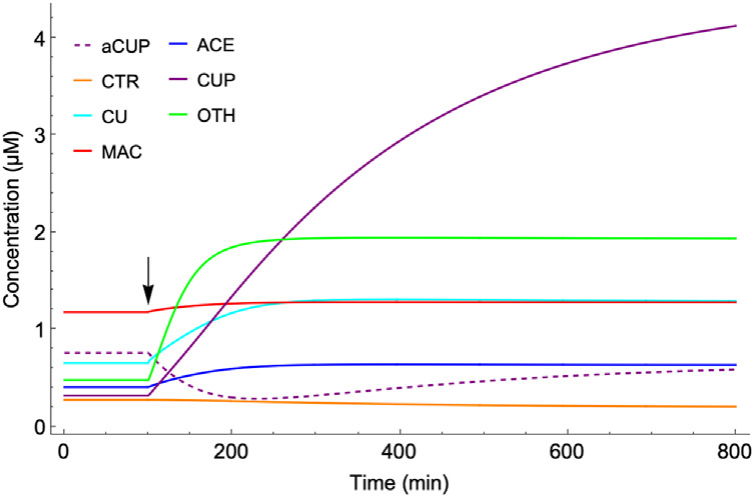
**Dynamical cellular response to increasing nutrient COPPER concentration from 10** **→** **250 μM at 100 min.**

The annealed dynamical system was perturbed extracellularly by abruptly increasing [COPPER] from 10 → 250 μM at *t* = 100 min (Fig. 5, arrow). The system responded by changing component concentrations as expected, attracting to a new steady-state. Many components responded within a single doubling-time (*t* = 210 min) but CUP required over 700 min to adjust. This is likely longer than would be observed experimentally; again, the rates of recovery reflect the assigned rates of reaction, and they should be fine-tuned as more experimental results become available.

### Steady-state effects of changing nutrient [COPPER]

**Figure 6:**
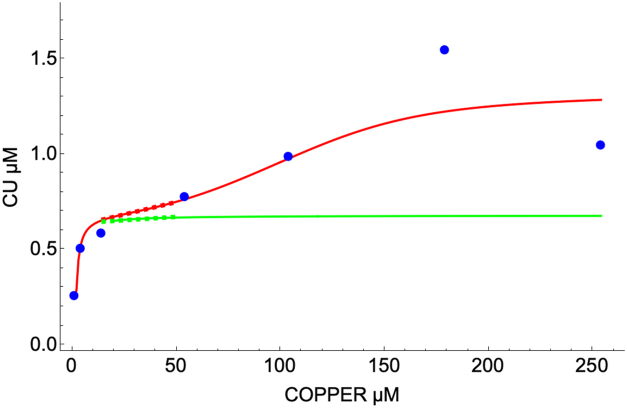
**Homeostatic effectiveness as judged by the change in the steady-state concentration of the labile Cu pool as nutrient COPPER concentrations was changed incrementally from 1** **→** **250 μM**. At each point, the plotted [CU] was obtained after an integration of *t* = 20,000 min. The *dashed line* indicates the relevant region for evaluating homeostatic effectiveness.

In these simulations, the *in silico* cell was grown on different nutrient [COPPER] concentrations, and the annealed dynamical system was allowed to respond long enough (*t* = 20,000 min) to reach steady state. For the seven conditions measure by Kim, the system generated the steady-state component concentrations listed in Table S8. However, the cell could grow on any fixed concentration of nutrient COPPER, yielding a plot of changing component concentrations, as shown for CU in Figure 6. The inverse-slope of the simulation line (the flatness) reflects the steady-state homeostatic effectiveness of the system. The region with the blue dashed line reflects the region (10 → 50 μM [COPPER]) in which the labile Cu pool CU changes minimally with a change of [COPPER]; in this case yielding a slope of 2.6 nM change in [CU] for each 1.0 μM change in [COPPER]. Such slopes could be used to compare the homeostatic effectiveness of various mechanisms, with perfect regulation affording a slope of zero.

The range of [CU] evident from this plot represented the “window” in which the labile copper pool is regulated, as predicted by the model. These limits are indicated by the arrows in Figure 3, bottom panel. Surprisingly, they are ∼ 3 orders of magnitude “tighter” than the window suggested by Wegner *et al.* (10).

### Response to “deleting” the CUIN2 importer

**Figure 7:**
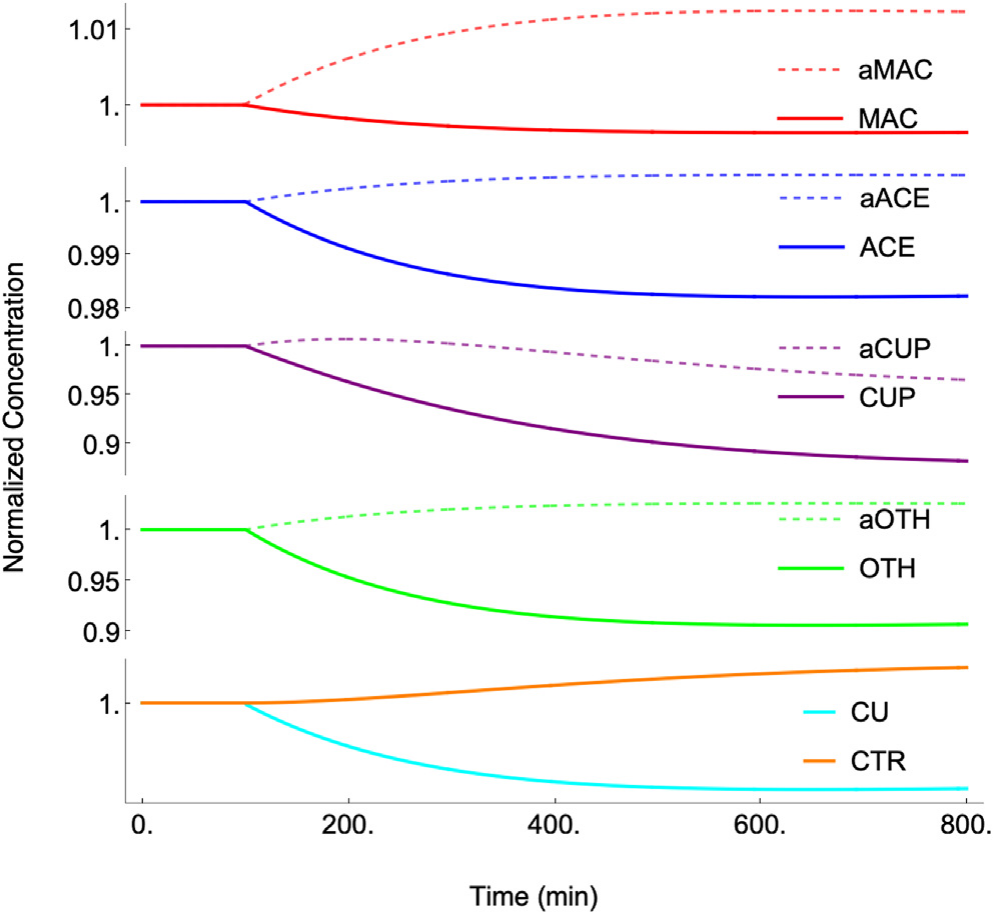
**Attraction of the dynamical system to a new steady-state after deleting the CUIN2 import pathway at *t* = 100 min**. In this simulation, [COPPER] was 10 μM.

The dynamical *in silico* cell can respond to deleting or overexpressing any gene/protein within the reaction network. This can be done by adjusting the rate-constant for the reaction involving the selected gene/protein. To illustrate this, we set the CUIN2 importer rate-constant to zero. Doing this caused the cell to become attracted to a different steady-state (Fig. 7) which is tantamount in actual cells to generating the mutant cell phenotype. The deletion was made at *t* = 100 min, and the response was immediate. As expected, less Cu entered the cell, causing a decline in CU, OTH, CUO, ACE, and MAC, and an increase in the apo forms. The increased level of aMAC stimulated the expression of CTR, but this response was insufficient to fully counter the effect of the primary “mutation”.

We also evaluated the homeostatic effectiveness of the mutant cell, and compared it to that of the WT cell. The cellular Cu concentrations of the steady-state component concentrations at increasing [COPPER] are plotted in Figure 2, bottom panel. Without the second importer, only about 10 μM Cu could enter the mutant cell. We also perturbed the mutant cell by doubling [CU], identical to how we treated the WT cell in Figure 4. The recovery rate was similar, likely because the intercellular homeostatic mechanism is unchanged when CUIN2 is deleted. We also perturbed [COPPER] as with the WT cell in Figure 5. In this case the recovery was faster with the mutant cell, but the concentration of copper entering the cell was much less. Finally, we calculated the steady-state [CU] as [COPPER] increased. In this case (Fig. 6, green line), [CU] changed nearly fourfold less than the WT cell, affording a slope of just 0.70 nM [CU] per μM change in [COPPER] in the dashed line region. Also, at higher [COPPER], [CU] was essentially invariant (plot nearly flat). We conclude that the homeostatic regulation of copper actually *improves* when CUIN2 is absent. We explain this nonintuitive result below.

## Discussion

### The insignificance of “free” copper

This ambiguous phrase technically only applies to copper ions that are not coordinated to any ligands. Such ions exist in the gas phase but not in aqueous solutions. The same phrase might refer to copper ions in solution that are exclusively coordinated by rapidly exchanging waters and/or hydroxide ions. A more accurate name for such species is aqueous copper ions. Historically, “free” copper has sometimes referred to copper ions that are coordinated by ligands other than water and that are either labile, chelatable, or exchangeable. The conclusion of Rae *et al.* (17) and others that cells contain no *aqueous* Cu is correct, but the same conclusion does not hold for labile copper. Here, we assumed that labile copper corresponds to the low-molecular-mass nonproteinaceous copper complexes detected by Kim *et al.* (13). The collective cellular concentrations of these complexes are about 11 orders of magnitude higher than reported for “free” Cu by earlier investigators. One reason for this was that all *K*_*d*_ values reported for Cu-binding proteins were scaled or normalized to aqueous Cu binding (*e.g.* Equation 1). However, since there is no aqueous Cu in the cell, we propose that they should be scaled to labile Cu, at least for the purpose of understanding copper homeostasis. We realize that this would not be possible until *K*_*d*_ values for the labile pool complexes are known. We recalibrated the data of Wegner *et al.* (10) so that we could use them in model development.

### Strategies to constrain the ODE system

As is typical of ODE-based models of biochemical processes, sufficient information required to solve this system uniquely was either unavailable or unreliable. Required information included the following: (a) the biochemical reaction network; (b) the rate-law expression for each reaction in the network; (c) component concentrations; (d) rate-constants; (e) Michaelis–Menten *K*_*m*_ constants; (f) the number of coppers that bind each protein; (g) the occupancy of copper binding for each protein; and (h) whether the binding of Cu to each protein is cooperative. However, ***all*** of these aspects must be decided upon before the ODE system can be integrated. Here, we relied on the published literature including: (a) the copper concentrations of Kim (13); (b) the proteomics data of Ho *et al.* (12); (c) the MAC/ACE deactivation/activation data of Wegner *et al.* (10); (d) the exponential growth rate of the cell (30); and (e) the *K*_*m*_ values of Lin *et al.* (26) and Hassett *et al.* (27). We constrained the model to be both physically possible and realistic. The basic pathways approach ensured that all rates were non-negative. Rates were selected as being independent or dependent to maximize reliance on more reliable information and to minimize reliance on unknowns. The solved system was in quantitative agreement with the results of Kim *et al.* (13). It afforded Cu-protein concentrations reasonably near to published proteomics values (12), and it simulated the results of Wegner *et al.* (10) after recalibration. Moreover, the dynamical system was attracted to a stable steady-state during the self-annealing process. Starting from the annealed steady-state, the system recovered from perturbations as required for any viable homeostatic mechanism. These results and behaviors demonstrate the viability of the model. The model makes a multitude of testable predictions *e.g.* regarding the effects of deleting any gene/protein/reaction in the network. Imperfect but well developed and documented models such as ours can still provide valid insights and advances (31). Moreover, the model makes an important prediction regarding the second importer.

### Prediction of a second importer

Our initial model included a single Cu importer with characteristics of CTR1 in which the expression level was controlled by MAC, as is established (23, 25, 32). However, this feature caused Cu import to decline as aMAC became metallated, consistent with the regulatory “window” proposed by Wegner *et al.* (10). However, this was not the behavior observed by Kim who found that Cu continues to enter the cell as nutrient [COPPER] increases. This puzzling discrepancy was resolved by adding a second Cu importer whose expression level was *not* controlled by aMAC. The success of this addition also suggested a biological explanation for the observed unusual homeostatic mechanism (*i.e.* the unregulated import of Cu while simultaneously expressing CUP1 to bind the excess imported Cu), namely that the second importer was not specific for Cu but that it also imported another essential metal ion. We suggest that the cell tolerates excessive Cu import (and tolerates a decline in homeostatic effectiveness) so that it can import another essential metal. We caution that this behavior may only occur in cells that are respiring on minimal media.

What is the second importer? Kim *et al.* (13) collected a series of Mössbauer spectra of whole cells grown on minimal media (refer to Figure 6 in reference (13)). The media for each sample were supplemented with 40 μM ^57^Fe^III^ citrate. Cells were grown with different levels of CuSO_4_ supplementation, mirroring the *M* titration. The spectrum of Cu-deficient cells (grown with chelator and without CuSO_4_) was dominated by a feature due to high-spin S = 5/2 Fe^III^ species shown previously to be located in vacuoles (33). Its presence indicates that the cell “felt” iron-replete as is typical for cells grown in such media (34). Unexpectedly, spectra of whole cells grown on higher concentrations of CuSO_4_ showed a decline in vacuolar Fe^III^ and a general reduction in spectral intensity; at 250 μM CuSO_4_, no vacuolar iron was evident. This suggests that cells grown on increasing levels of CuSO_4_ “felt” less iron-replete—even though the media contained the same concentration of ^57^Fe^III^ citrate. We hypothesize that under these growth conditions, iron enters through an importer that also imports Cu, specifically FET4, such that the two metals compete for entry. FET4 imports Cu along with other metals such as iron, as characterized by Kosman and Culotta (4, 26, 27).

Parenthetically, another intense feature in the Mössbauer spectra was a quadruple doublet due primarily to S = 0 [Fe_4_S_4_]^2+^ clusters. The intensity of this feature was largely invariant across the series. Iron-sulfur clusters are easily inactivated by aqueous Cu and some labile Cu species (35), so the integrity of these clusters implies (surprisingly) that the increasing level of labile pool copper in the cell (roughly 0.27 → 1.2 μM as measured by Kim) is not harmful to these clusters as a group. This suggests that the members of the labile Cu pool are coordinated by relatively tight-binding ligands, and that this limits the toxic effects of aqueous Cu. The reducing conditions of the cell may also maintain pool Cu in the 1+ state and thus prevent redox cycling, which may further limit its toxic effects.

### Increasing rate constants in four reactions

The rate-constants of 21 of 25 reactions essentially remained invariant as nutrient COPPER levels increased; this suggests that the assumed rate-law expressions were approximately accurate. The remaining four rate-constants increased significantly (and nonlinearly) as COPPER levels increased, implying that the assumed rate-law lacked an important influence. Most of these reactions involved CUP—its biosynthesis and metallation, along with the increased import of copper to which aCUP bound (through CUIN2). The rates of these reactions are most likely controlled by protein expression levels, which are in turn controlled by transcription factors that are sensitive to labile Cu pool levels. We used logistical functions as simple surrogates for these uncertain processes. These functions required sensitivity factors *n* > 0, indicating feedforward rather than feedback. For [CU] < *Sen*_*sp*_, the functions increased nonlinearly as [CU] → *Sen*_*sp*_, which fits the observed behavior. For [CU] > *Sen*_*sp*_ and increasing, the function plateaus. Thus, the model does not predict that cells would continue to increase COPPER import indefinitely as [COPPER] increases beyond 250 μM.

### Evaluating homeostatic effectiveness

Introductory sections of papers on cellular metal trafficking routinely emphasize the essentiality of the metal along with the dangers that the metal poses to the cell, with the conclusion being that homeostatic regulation of the metal must be “tight”. This term has been used subjectively and qualitatively, but ODE-based mathematical models like the one presented here allow homeostatic regulatory effectiveness to be evaluated quantitatively and objectively. Our analysis suggests numerous evaluation criteria. Certainly, the system must return to its original state after an internal perturbation of a component concentration; those that fail in this regard should not be categorized as homeostatic mechanisms. The more extreme the perturbation that can be tolerated, the more robust the regulation. The steady-state concentration of regulated components in a perfectly regulated system will be unaffected by changes in the extracellular environment (*i.e.* nutrient concentrations). Finally, the wider the range of extracellular (nutrient) changes that generate little or no changes in intracellular components, the more effective the homeostatic mechanism. The use of these criteria in evaluating future models of homeostatic regulation would allow for more quantitative and objective comparisons between models, as well as more direct connections to experimental results.

## Experimental procedures

No experiments were performed in this study. Most computations were performed using code written in *Mathematica* (https://www.wolfram.com/mathematica/), included in Supplemental Information.

## Data availability

All data are contained within the manuscript and SI.

## Supporting information

This article contains supporting information (12, 13, 36, 37, 38, 39).

## Conflict of interest

The authors declare that they have no conflicts of interest with the contents of this article.

## Supporting information

Supporting information

DulaneyCopperModelCode
