# Supplementary material for: A kinetic model of copper homeostasis in Saccharomyces cerevisiae
Source: J Biol Chem. 2025 Jun 16;301(8):110368. doi: 10.1016/j.jbc.2025.110368 (PMC12309601; doi:10.1016/j.jbc.2025.110368)
Supplement: Supporting information [file mmc1.docx]

**Supplemental Information for…**

A kinetic model of copper homeostasis in *Saccharomyces cerevisiae*

Cade Dulaney, Jay R. Walton, and Paul A. Lindahl

Table of contents:

Page 2: Table S1: Copper-containing proteins and their concentrations in respiring S. cerevisiae.

Page 4: Table S2: Copper and protein concentrations and fractional occupancies associated with the Cu-bound groups in S. cerevisiae cells and model.

Page 5: Table S3: Model reactions, catalysts, and rate-law expressions.

Page 7: Table S4: The stoichiometric ***S*** matrix

Page 8: Table S5: The ***W*** matrix associated with the model.

Page 9: Figure S1: Description of basic pathways.

Page 11: Table S6: Steady-state reaction rates (μM/min)

Page 12: Table S7: Rate-constants (μM/min)

Page 13: Table S8: Kinetic parameters used for the dynamical system

**Table S1: Copper-containing proteins and their concentrations in respiring *S. cerevisiae*.** In most cases, “copies per cell” is the average values from Ho et al (12). and the *Saccharomyces Genome Database (https://www.yeastgenome.org/)*; see Table S1 of Kim et al. (13) for individual entries. Published copies-per-cell values were generally obtained by quantitative mass spectra of extracts of cells grown under fermenting conditions on YPD media and without Cu supplementation or Cu chelators. The model is of trafficking and regulation of copper in respiring cells (ignoring any cell wall contributions).

The estimated cellular [Cu] associated with each protein under fermenting conditions was calculated using the equation

$[Cu]_{cell}=\frac{\text{Protein} \text{copies}}{\text{cell}}\cdot\frac{\text{copper ions}}{\text{Protein}}\cdot\frac{1 mol}{6.022\times10^{23} copies}\cdot\frac{1 cell}{42\times10^{-15} L}\cdot\frac{10^{6} \mu mol}{mol}$.

Ccs1 and Sod1 concentrations are taken from Rae et al (17). The Crs5 concentration was estimated to be ~20% of the Cup1 concentration. The Ctr3 concentration was excluded because it is not expressed in most laboratory yeast strains (36). Cox1 and Cox2 concentrations refer to respiring cells, taken from Morales et al. (37). Assumed fractional volumes for cytosol, mitochondria, nucleus, vacuoles, and endoplasmic reticula were 0.64, 0.10, 0.15, 0.082, and 0.024, respectively; see Table S2 of Lindahl and Vali (38). For proteins with dual localization, 50% were assumed to be in each compartment except for Ccs1 and Sod1, 90% of which were assumed to be in cytosol and 10% in mitochondria (39). The Cu concentrations in the table were calculated assuming the listed stoichiometry and that 30% of the proteins are metallated as estimated for M10 conditions by Kim et al. (13).

| Standard Name | Systematic  Name | Copies per cell  (monomers) | [Protein]_cell_  (µM) | Assumed Coppers per  monomer | [Cu]_cell_ (µM) | Cellular location(s) | Soluble (s) or membrane-bound (m) | [Protein]_local_  (uM) | [Cu]_local_ |
| --- | --- | --- | --- | --- | --- | --- | --- | --- | --- |
| Atx1 | YNL259C | 8200 | 0.32 | 1 | 0.098 | cytosol | s | 0.53 | 0.16 |
| Ccs1 | YMR038C | 10,000 | 0.39 | 1 | 0.12 | cytosol | s | 0.57 | 0.17 |
| Cox17 | YLL009C | 16,000 | 0.64 | 1 | 0.19 | cytosol | s | 0.52 | 0.16 |
| Cox23 | YHR116W | 2500 | 0.10 | 1 | 0.030 | cytosol | s | 0.081 | 0.024 |
| Crs5 | YOR031W | --- | 0.050 | 8 | 0.12 | cytosol | s | 0.041 | 0.098 |
| Cup1-1 | YHR053C | 3500 | 0.14 | 8 | 0.33 | cytosol | s | 0.11 | 0.27 |
| Cup1-2 | YHR055C | 2500 | 0.10 | 8 | 0.24 | cytosol | s | 0.081 | 0.20 |
| Sod1 | YJR104C | 60,000 | 2.4 | 1 | 0.71 | cytosol | s | 3.5 | 1.0 |
|  |  |  |  |  |  |  |  |  | **2.1 µM soluble cytosolic Cu.** |
| Ctr1 | YPR124W | 4500 | 0.18 | 0 | 0 | plasma membrane | m | 0.29 | 0 |
| Ctr3 | YLR411W | --- | --- | 0 | 0 | plasma membrane | m | --- | 0 |
| Fet3 | YMR058W | 6900 | 0.27 | 4 | 0.33 | plasma membrane | m | 0.44 | 0.53 |
|  |  |  |  |  |  |  |  |  | **0.53**  **µM plasma**  **Membrane-bound Cu** |
| Cox1 | Q0045 | 2400 | 3 | 1 | 0.90 | mitochondria | m | 30 | 9.0 |
| Cox2 | Q0250 | 4500 | 3 | 2 | 1.8 | mitochondria | m | 30 | 18 |
| Cox11 | YPL132W | 1800 | 0.069 | 1 | 0.021 | mitochondria | s | 0.69 | 0.21 |
| Cox19 | YLL018C-A | 4100 | 0.16 | 1 | 0.049 | mitochondria | s | 1.6 | 0.49 |
| Sco1 | YBR037C | 2600 | 0.10 | 1 | 0.031 | mitochondria | m | 1.0 | 0.31 |
| Cox17 |  | (Dual) |  |  |  | mitochondria | s | 3.2 | 3.8 |
| Cox23 |  | (Dual) |  |  |  | mitochondria | s | 0.50 | 0.15 |
| Cup1-1 |  | (Dual) |  |  |  | mitochondria | s | 0.70 | 1.7 |
| Cup1-2 |  | (Dual) |  |  |  | mitochondria | s | 0.50 | 1.2 |
| Sod1 |  | (Dual; 10% m) |  |  |  | mitochondria | s | 3.4 | 1.0 |
|  |  |  |  |  |  |  |  |  | **36 µM**  **Mitochondrial Cu** |
|  |  |  |  |  |  |  |  |  |  |
| Ace1 | YGL166W | 1500 | 0.058 | 4 | 0.070 | nucleus | s | 0.46 | 0.56 |
| Mac1 | YMR021C | 1200 | 0.048 | 4 | 0.12 | nucleus | s | 0.38 | 0.46 |
|  |  |  |  |  |  |  |  |  | **1.0 µM**  **Nuclear Cu** |
|  |  |  |  |  |  |  |  |  |  |
| Ccc2 | YDR270W | 1100 | 0.043 | 0 | 0 | Golgi (ER) | M | 2.9 (assume ER) | 0 |
|  |  |  |  |  |  |  |  |  | **0 µM Cu in ER** |
|  |  |  |  |  |  |  |  |  |  |
| Ctr2 | YHR175W | 2400 | 0.096 | 1 | 0.096 | vacuoles | M | 1.4 | 0 |
| Fet5 | YFL041W | 4000 | 0.16 | 4 | 0.19 | vacuoles | M | 2.4 | 2.8 |
|  |  |  |  |  |  |  |  |  | **2.8 µM**  **Vacuolar Cu** |
|  | | | | | | Protein-bound Cu in cell: 2.6*0.64 + 36*0.1 + 1.0*0.15 + 2.8*0.082 = 5.9 µM | | | |

**Table S2** C**opper and protein concentrations and fractional occupancies associated with the Cu-bound groups in *S. cerevisiae* cells and model**. Cells were respiring in minimal media with 7 different levels of copper supplementation (13). Fractional occupancies in parentheses were obtained by interpolating between measured values. Concentrations of proteins are calculated from E6. All concentrations are in μM.

| **Media in M titration (from Kim)** | MBCS | M0 | M10 | M50 | M100 | M175 | M250 |
| --- | --- | --- | --- | --- | --- | --- | --- |
| Estimated [COPPER] in media | 1.0 | 4.0 | 14 | 54 | 100 | 180 | 250 |
| C+X group = CUP | 0.14 | 0.27 | 2.7 | 6.5 | 16 | 23 | 46 |
| O+S group = OTH | 0.27 | 0.27 | 0.67 | 0.97 | 1.8 | 2.3 | 2.7 |
| W+T group = CU | 0.26 | 0.51 | 0.59 | 0.78 | 0.99 | 1.6 | 1.0 |
| Total [Cu]cyt (sum of groups) | 0.67 | 1.1 | 4.0 | 8.2 | 19 | 27 | 49 |
|  |  |  |  |  |  |  |  |
| **Fractional occupancy of copper.** | 0.10 | (0.20) | 0.30 | (0.47) | (0.64) | 0.80 | (0.90) |
|  |  |  |  |  |  |  |  |
| **From proteomics (protein concentrations in Table S1)** |  |  |  |  |  |  |  |
| CUP = (Cup1-1 + Cup1-2 + Crs5) |  |  | 0.24 |  |  |  |  |
| OTH = (Atx1 + Ccs1 + Cox17 + Cox23 + Sod1) |  |  | 5.2 |  |  |  |  |
| MAC (in nucleus) |  |  | 1.5 |  |  |  |  |
| ACE (in nucleus) |  |  | 1.9 |  |  |  |  |
| CTR |  |  | 0.29 |  |  |  |  |
|  |  |  |  |  |  |  |  |
| **Estimated protein concentrations (for model, from E8)** |  |  |  |  |  |  |  |
| [CUP]tot = [CUP] + [aCUP] | 0.18 | 0.17 | 1.15 | 1.7 | 3.2 | 3.6 | 6.3 |
| [OTH]tot = [OTH] + [aOTH] | 2.7 | 1.4 | 2.2 | 2.1 | 2.8 | 2.9 | 3.0 |
| [CU] | 0.26 | 0.51 | 0.59 | 0.78 | 0.99 | 1.6 | 1.0 |
|  |  |  |  |  |  |  |  |
| **MAC and ACE ratios (from Wegner re-plot)** |  |  |  |  |  |  |  |
| [aMAC]/[MAC]tot (protein ratio) | 0.34 | 0.26 | 0.24 | 0.21 | 0.18 | 0.15 | 0.18 |
| [ACE]/[ACE]tot (protein ratio) | 0.11 | 0.18 | 0.20 | 0.25 | 0.29 | 0.38 | 0.30 |
|  |  |  |  |  |  |  |  |
| **Initial protein concentrations used for model** |  |  |  |  |  |  |  |
| CU | 0.26 | 0.51 | 0.59 | 0.78 | 0.99 | 1.6 | 1.0 |
| aCUP | 0.16 | 0.14 | 0.80 | 0.91 | 1.1 | 0.72 | 0.63 |
| CUP | 0.018 | 0.034 | 0.34 | 0.81 | 2.0 | 2.9 | 5.7 |
| aOTH | 2.4 | 1.1 | 1.6 | 1.1 | 1.0 | 0.58 | 0.30 |
| OTH | 0.27 | 0.27 | 0.67 | 0.97 | 1.8 | 2.3 | 2.7 |
| aMAC | 0.53 | 0.40 | 0.37 | 0.32 | 0.28 | 0.22 | 0.28 |
| MAC | 1.0 | 1.1 | 1.2 | 1.2 | 1.3 | 1.3 | 1.3 |
| aACE | 1.7 | 1.5 | 1.5 | 1.4 | 1.3 | 1.2 | 1.3 |
| ACE | 0.20 | 0.34 | 0.38 | 0.46 | 0.54 | 0.70 | 0.56 |
| CTR | 0.42 | 0.32 | 0.29 | 0.26 | 0.22 | 0.18 | 0.22 |
| Total Cu (8CUP+OTH+4MAC+4ACE+CU) | 5.5 | 6.9 | 10 | 15 | 26 | 35 | 56 |
|  |  |  |  |  |  |  |  |
| **Optimized protein concentrations to which the model was attracted** |  |  |  |  |  |  |  |
| CU | 0.28 | 0.56 | 0.66 | 0.77 | 0.98 | 1.2 | 1.3 |
| aCUP | 0.13 | 0.55 | 0.76 | 0.96 | 1.0 | 0.73 | 0.66 |
| CUP | 0.01 | 0.14 | 0.32 | 0.72 | 2.2 | 4.1 | 4.5 |
| aOTH | 2.2 | 2.0 | 1.8 | 1.4 | 0.69 | 0.34 | 0.30 |
| OTH | 0.026 | 0.26 | 0.48 | 0.84 | 1.5 | 1.9 | 1.9 |
| aMAC | 0.51 | 0.38 | 0.35 | 0.32 | 0.29 | 0.26 | 0.25 |
| MAC | 1.0 | 1.2 | 1.2 | 1.2 | 1.2 | 1.3 | 1.3 |
| aACE | 1.6 | 1.5 | 1.4 | 1.4 | 1.3 | 1.2 | 1.3 |
| ACE | 0.22 | 0.36 | 0.41 | 0.46 | 0.54 | 0.62 | 0.63 |
| CTR | 0.40 | 0.30 | 0.28 | 0.26 | 0.23 | 0.20 | 0.20 |
| Total Cu (8CUP+OTH+4MAC+4ACE+CU) | 5.3 | 8.0 | 10 | 14 | 27 | 43 | 47 |

**Table S3: Model Reactions, catalysts, and rate-law expressions.** Forward and reverse directions for a reaction as separate reactions. For four reactions, the rate law expression was modified as shown.

| Number | Name | Reaction | Catalyst | Rate Law (or equilibrium expression) |
| --- | --- | --- | --- | --- |
| 01 | BAMAC | → aMAC |  | $BMAC=kBMAC$ |
| 02 | BAACE | → aACE |  | $BACE=kBACE$ |
| 03 | BAOTH | → aOTH |  | $BOTH=kBOTH$ |
| 04 | BACUP | → aCUP | ACE | $BCUP=kBCUP\left(\frac{[ACE]}{[ACE]_{M10}}\right)$  Modified: $BCUP=kBCUP^{*}\left(\frac{[ACE]}{[ACE]_{M10}}\right)\left(\frac{1}{1+e^{(n\cdot(Sen_{sp}-[CU]))}}\right)$  |
| 05 | BCTR | → CTR | aMAC | $BCTR=kBCTR\left(\frac{[aMAC]}{[aMAC]_{M10}}\right)$ |
| 06 | CUIN1 | COPPER → Cu | CTR | $CUIN1=kCUIN1\cdot\left(\frac{[COPPER]}{K_{m(1)}+[COPPER]}\right)\left(\frac{[CTR]}{[CTR]_{M10}}\right)$ |
| 07 | CUIN2 | COPPER → Cu |  | $CUIN2=kCUIN2\cdot\left(\frac{[COPPER]}{K_{m(2)}+[COPPER]}\right)$  Modified $CUIN2=kCUIN2*\cdot\left(\frac{[COPPER]}{K_{m(2)}+[COPPER]}\right)\left(\frac{1}{1+e^{(n\cdot(Sen_{sp}-[COPPER]))}}\right)$  |
| 08 | MMACF | aMAC + 4Cu → MAC |  | $MMACF=kMMACF\left(\frac{[aMAC]}{[aMAC]_{M10}}\right)\left(\frac{[Cu]}{[Cu]_{M10}}\right)^{0.62}$ |
| 09 | MMACR | MAC → aMAC + 4Cu |  | $MMACR=kMMACR\left(\frac{[MAC]}{[MAC]_{M10}}\right)$ |
| 10 | MACEF | aACE + 4Cu →ACE |  | $MACEF=kMACEF\cdot\left(\frac{[aACE]}{[aACE]_{M10}}\right)\left(\frac{[Cu]}{[Cu]_{M10}}\right)^{0.9}$ |
| 11 | MACER | ACE →aACE + 4Cu |  | $MACER=kMACER\left(\frac{[ACE]}{[ACE]_{M10}}\right)$ |
| 12 | MCUPF | aCUP + 8Cu →CUP |  | $MCUPF=kMCUPF\cdot\left(\frac{[aCUP]}{[aCUP]_{M10}}\right)\left(\frac{[CU]}{[CU]_{M10}}\right)^{1.1}$  Modified $MCUPF=kMCUPF^{*}\cdot\left(\frac{[aCUP]}{[aCUP]_{M10}}\right)\left(\frac{[CU]}{[CU]_{M10}}\right)^{1.1}\left(\frac{1}{1+e^{(n\cdot(Sen_{sp}-[CU]))}}\right)$  |
| 13 | MCUPR | CUP → aCUP + 8Cu |  | $MCUPR=kMCUPR\left(\frac{[CUP]}{[CUP]_{M10}}\right)$ |
| 14 | MOTHF | aOTH + Cu →OTH |  | $MOTHF=kMOTHF\cdot\left(\frac{[aOTH]}{[aOTH]_{M10}}\right)\left(\frac{[Cu]}{[Cu]_{M10}}\right)$  modified $MOTHF=kMOTHF^{*}\cdot\left(\frac{[aOTH]}{[aOTH]_{M10}}\right)\left(\frac{[Cu]}{[Cu]_{M10}}\right)\left(\frac{1}{1+e^{(n\cdot(Sen_{sp}-[CU]))}}\right)$  |
| 15 | MOTHR | OTH → aOTH + Cu |  | $MOTHR=kMOTHR\left(\frac{[OTH]}{[OTH]_{M10}}\right)$ |
| 16 | DAMAC | aMAC → |  | $DAMAC=\alpha_{cell}\cdot[aMAC]$ |
| 17 | DMAC | MAC → |  | $DMAC=\alpha_{cell}\cdot[MAC]$ |
| 18 | DAACE | aACE→ |  | **$DAACE=\alpha_{cell}\cdot[aACE]$** |
| 19 | DACE | ACE→ |  | **$DACE=\alpha_{cell}\cdot[ACE]$** |
| 20 | DCTR | CTR → |  | $DCTR=\alpha_{cell}\cdot[CTR]$ |
| 21 | DACUP | aCUP → |  | $DACUP=\alpha_{cell}\cdot[aCUP]$ |
| 22 | DCUP | CUP → |  | $DCUP=\alpha_{cell}\cdot[CUP]$ |
| 23 | DAOTH | aOTH → |  | $DAOTH=\alpha_{cell}\cdot[aOTH]$ |
| 24 | DOTH | OTH → |  | $DOTH=\alpha_{cell}\cdot[OTH]$ |
| 25 | DCU | CU → |  | $DCU=\alpha_{cell}\cdot[CU]$ |

**Table S4. The stoichiometric “S” matrix**

|  | BAMAC | BAACE | BAOTH | BACUP | BCTR | CUIN1 | CUIN2 | MMACF | MMACR | MACEF | MACER | MCUPF | MCUPR | MOTHF | MOTHR |
| --- | --- | --- | --- | --- | --- | --- | --- | --- | --- | --- | --- | --- | --- | --- | --- |
| aMAC | 1 | 0 | 0 | 0 | 0 | 0 | 0 | -1 | 1 | 0 | 0 | 0 | 0 | 0 | 0 |
| aACE | 0 | 1 | 0 | 0 | 0 | 0 | 0 | 0 | 0 | -1 | 1 | 0 | 0 | 0 | 0 |
| aOTH | 0 | 0 | 1 | 0 | 0 | 0 | 0 | 0 | 0 | 0 | 0 | 0 | 0 | -1 | 1 |
| aCUP | 0 | 0 | 0 | 1 | 0 | 0 | 0 | 0 | 0 | 0 | 0 | -1 | 1 | 0 | 0 |
| CTR | 0 | 0 | 0 | 0 | 1 | 0 | 0 | 0 | 0 | 0 | 0 | 0 | 0 | 0 | 0 |
| CU | 0 | 0 | 0 | 0 | 0 | 1 | 1 | -4 | 4 | -4 | 4 | -8 | 8 | -1 | 1 |
| MAC | 0 | 0 | 0 | 0 | 0 | 0 | 0 | 1 | -1 | 0 | 0 | 0 | 0 | 0 | 0 |
| ACE | 0 | 0 | 0 | 0 | 0 | 0 | 0 | 0 | 0 | 1 | -1 | 0 | 0 | 0 | 0 |
| CUP | 0 | 0 | 0 | 0 | 0 | 0 | 0 | 0 | 0 | 0 | 0 | 1 | -1 | 0 | 0 |
| OTH | 0 | 0 | 0 | 0 | 0 | 0 | 0 | 0 | 0 | 0 | 0 | 0 | 0 | 1 | -1 |

**…**

Continued…

|  | DAMAC | DAACE | DAOTH | DACUP | DCTR | DCU | DMAC | DACE | DCUP | DOTH |
| --- | --- | --- | --- | --- | --- | --- | --- | --- | --- | --- |
| aMAC | -1 | 0 | 0 | 0 | 0 | 0 | 0 | 0 | 0 | 0 |
| aACE | 0 | -1 | 0 | 0 | 0 | 0 | 0 | 0 | 0 | 0 |
| aOTH | 0 | 0 | -1 | 0 | 0 | 0 | 0 | 0 | 0 | 0 |
| aCUP | 0 | 0 | 0 | -1 | 0 | 0 | 0 | 0 | 0 | 0 |
| CTR | 0 | 0 | 0 | 0 | -1 | 0 | 0 | 0 | 0 | 0 |
| CU | 0 | 0 | 0 | 0 | 0 | -1 | 0 | 0 | 0 | 0 |
| MAC | 0 | 0 | 0 | 0 | 0 | 0 | -1 | 0 | 0 | 0 |
| ACE | 0 | 0 | 0 | 0 | 0 | 0 | 0 | -1 | 0 | 0 |
| CUP | 0 | 0 | 0 | 0 | 0 | 0 | 0 | 0 | -1 | 0 |
| OTH | 0 | 0 | 0 | 0 | 0 | 0 | 0 | 0 | 0 | -1 |

…

**Table S5: The *W* matrix associated with the model.**

|  | BP01 | BP02 | BP03 | BP04 | BP05 | BP06 | BP07 | BP08 | BP09 | BP10 | BP11 | BP12 | BP13 | BP14 | BP15 |
| --- | --- | --- | --- | --- | --- | --- | --- | --- | --- | --- | --- | --- | --- | --- | --- |
| BMAC | 1 | 0 | 0 | 0 | 0 | 0 | 0 | 1 | 1 | 0 | 0 | 0 | 0 | 0 | 0 |
| BACE | 0 | 1 | 0 | 0 | 0 | 0 | 0 | 0 | 0 | 1 | 1 | 0 | 0 | 0 | 0 |
| BOTH | 0 | 0 | 1 | 0 | 0 | 0 | 0 | 0 | 0 | 0 | 0 | 0 | 0 | 1 | 1 |
| BCUP | 0 | 0 | 0 | 1 | 0 | 0 | 0 | 0 | 0 | 0 | 0 | 1 | 1 | 0 | 0 |
| BCTR | 0 | 0 | 0 | 0 | 1 | 0 | 0 | 0 | 0 | 0 | 0 | 0 | 0 | 0 | 0 |
| CUIN1 | 0 | 0 | 0 | 0 | 0 | 1 | 0 | 4 | 4 | 4 | 4 | 8 | 8 | 1 | 1 |
| CUIN2 | 0 | 0 | 0 | 0 | 0 | 0 | 1 | 0 | 0 | 0 | 0 | 0 | 0 | 0 | 0 |
| MMACF | 0 | 0 | 0 | 0 | 0 | 0 | 0 | 1 | 1 | 0 | 0 | 0 | 0 | 0 | 0 |
| MMACR | 0 | 0 | 0 | 0 | 0 | 0 | 0 | 0 | 1 | 0 | 0 | 0 | 0 | 0 | 0 |
| MACEF | 0 | 0 | 0 | 0 | 0 | 0 | 0 | 0 | 0 | 1 | 1 | 0 | 0 | 0 | 0 |
| MACER | 0 | 0 | 0 | 0 | 0 | 0 | 0 | 0 | 0 | 0 | 1 | 0 | 0 | 0 | 0 |
| MCUPF | 0 | 0 | 0 | 0 | 0 | 0 | 0 | 0 | 0 | 0 | 0 | 1 | 1 | 0 | 0 |
| MCUPR | 0 | 0 | 0 | 0 | 0 | 0 | 0 | 0 | 0 | 0 | 0 | 0 | 1 | 0 | 0 |
| MOTHF | 0 | 0 | 0 | 0 | 0 | 0 | 0 | 0 | 0 | 0 | 0 | 0 | 0 | 1 | 1 |
| MOTHR | 0 | 0 | 0 | 0 | 0 | 0 | 0 | 0 | 0 | 0 | 0 | 0 | 0 | 0 | 1 |
| DAMAC | 1 | 0 | 0 | 0 | 0 | 0 | 0 | 0 | 1 | 0 | 0 | 0 | 0 | 0 | 0 |
| DAACE | 0 | 1 | 0 | 0 | 0 | 0 | 0 | 0 | 0 | 0 | 1 | 0 | 0 | 0 | 0 |
| DAOTH | 0 | 0 | 1 | 0 | 0 | 0 | 0 | 0 | 0 | 0 | 0 | 0 | 0 | 0 | 1 |
| DACUP | 0 | 0 | 0 | 1 | 0 | 0 | 0 | 0 | 0 | 0 | 0 | 0 | 1 | 0 | 0 |
| DCTR | 0 | 0 | 0 | 0 | 1 | 0 | 0 | 0 | 0 | 0 | 0 | 0 | 0 | 0 | 0 |
| DCU | 0 | 0 | 0 | 0 | 0 | 1 | 1 | 0 | 4 | 0 | 4 | 0 | 8 | 0 | 1 |
| DMAC | 0 | 0 | 0 | 0 | 0 | 0 | 0 | 1 | 0 | 0 | 0 | 0 | 0 | 0 | 0 |
| DACE | 0 | 0 | 0 | 0 | 0 | 0 | 0 | 0 | 0 | 1 | 0 | 0 | 0 | 0 | 0 |
| DCUP | 0 | 0 | 0 | 0 | 0 | 0 | 0 | 0 | 0 | 0 | 0 | 1 | 0 | 0 | 0 |
| DOTH | 0 | 0 | 0 | 0 | 0 | 0 | 0 | 0 | 0 | 0 | 0 | 0 | 0 | 1 | 0 |

**
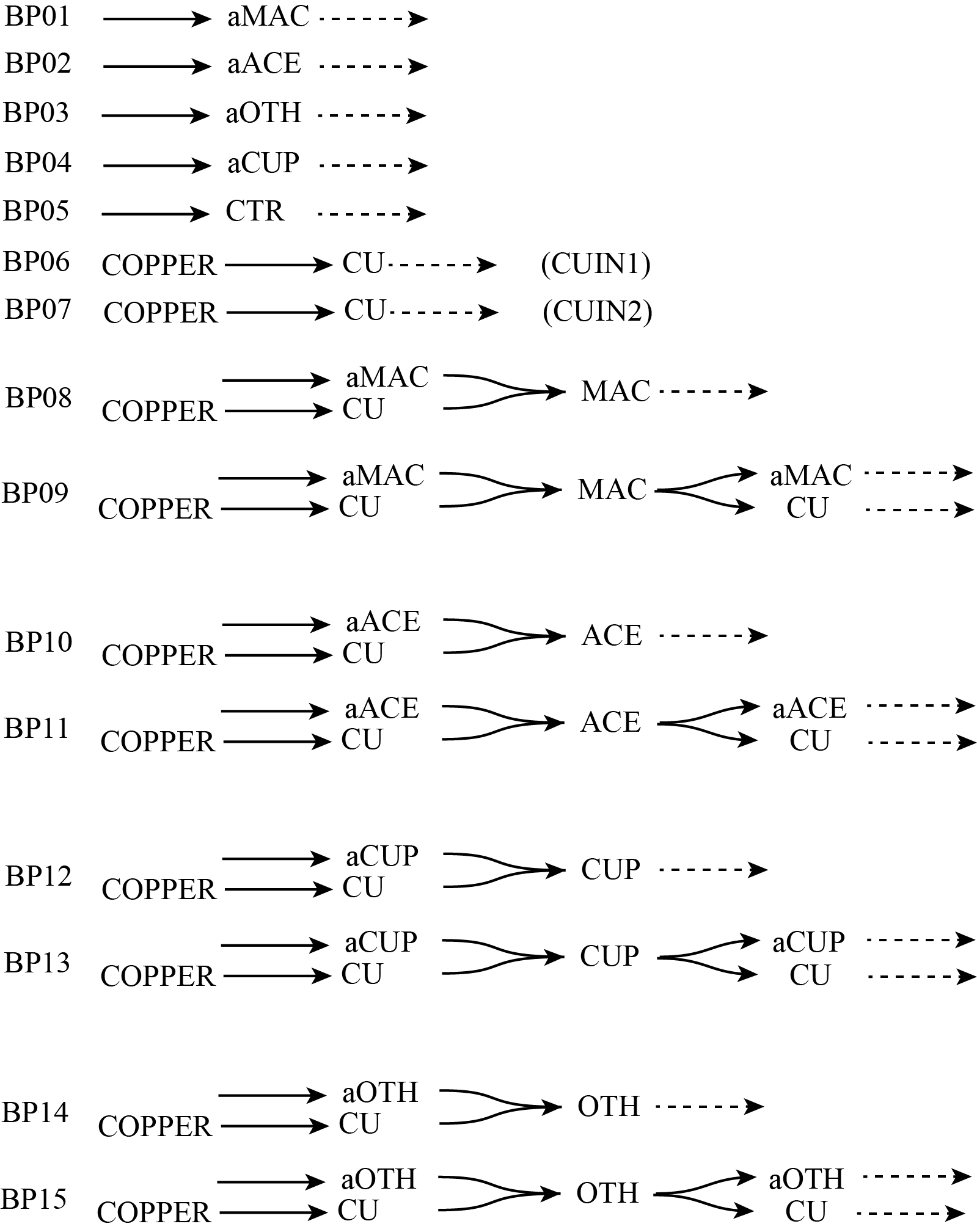
Figure S1: Description of Basic Pathways.** The reaction network used in this study has 15 basic pathways, indicated BP01 → BP15. Most basic pathways specify a stoichiometric flow through the reaction network. They often start with a specific nutrient or group of nutrients and terminate at one or more specific components in the model, followed by the dilution of those components. They also specify the specific reactions used to connect start to finish. In the drawings below, dashed lines indicate dilution reactions. BP01 – BP05 are the biosynthetic pathways for the indicated 5 proteins in their apo forms. No nutrients are at the start of the pathway because none were included in the corresponding reaction (assumed to be invariant under all conditions). BP06 and BP07 are the pathways from nutrient COPPER to the labile Cu pool CU, with BP06 going through CUIN1 and BP07 going through CUIN2. BP08 describes the pathway from nutrients to the metallated form of MAC. BP10, BP12, and BP14 are similar pathways leading to the metallated forms of ACE, CUP, and OTH, respectively. BP09 is the pathway terminating at the unmetallated form of MAC (aMAC) and CU, but passing through the metallated form of MAC. BP11, BP13, and BP15 are similar pathways leading to the unmetallated forms of ACE, CUP, and OTH.

**Table S6: Steady-state reaction rates (μM/min)**

| Titration→  Reaction↓ | MBCS | M0 | M10 | M50 | M100 | M175 | M250 |
| --- | --- | --- | --- | --- | --- | --- | --- |
| BAMAC | 0.0051 | 0.0051 | 0.0051 | 0.0051 | 0.0051 | 0.0051 | 0.0051 |
| BAACE | 0.0062 | 0.0061 | 0.0062 | 0.0062 | 0.0062 | 0.0062 | 0.0062 |
| BAOTH | 0.0090 | 0.0045 | 0.0074 | 0.0069 | 0.0095 | 0.0097 | 0.010 |
| BACUP | 0.00058 | 0.00056 | 0.0038 | 0.0057 | 0.011 | 0.012 | 0.021 |
| BCTR | 0.0014 | 0.0010 | 0.00097 | 0.00085 | 0.00075 | 0.00059 | 0.00073 |
| CUIN1 | 0.018 | 0.023 | 0.033 | 0.032 | 0.029 | 0.023 | 0.028 |
| CUIN2 | 0.0 | 0.00010 | 0.00055 | 0.017 | 0.059 | 0.093 | 0.16 |
| MMACF | 0.34 | 0.38 | 0.39 | 0.41 | 0.42 | 0.44 | 0.42 |
| MMACR | 0.33 | 0.38 | 0.39 | 0.40 | 0.42 | 0.44 | 0.42 |
| MACEF | 0.068 | 0.11 | 0.13 | 0.16 | 0.18 | 0.24 | 0.19 |
| MACER | 0.068 | 0.11 | 0.13 | 0.15 | 0.18 | 0.23 | 0.19 |
| MCUPF | 0.0059 | 0.011 | 0.12 | 0.27 | 0.69 | 0.96 | 1.9 |
| MCUPR | 0.0058 | 0.011 | 0.11 | 0.27 | 0.68 | 0.95 | 1.9 |
| MOTHF | 0.091 | 0.091 | 0.22 | 0.33 | 0.61 | 0.78 | 0.92 |
| MOTHR | 0.090 | 0.090 | 0.22 | 0.32 | 0.60 | 0.78 | 0.91 |
| DAMAC | 0.0018 | 0.0013 | 0.0012 | 0.0011 | 0.00094 | 0.00075 | 0.00092 |
| DAACE | 0.0055 | 0.0050 | 0.0049 | 0.0047 | 0.0044 | 0.0038 | 0.0043 |
| DAOTH | 0.0081 | 0.0036 | 0.0052 | 0.0036 | 0.0034 | 0.0019 | 0.0010 |
| DACUP | 0.00052 | 0.00045 | 0.0027 | 0.0030 | 0.0038 | 0.0024 | 0.0021 |
| DCTR | 0.0014 | 0.0010 | 0.00097 | 0.00085 | 0.00075 | 0.00059 | 0.00073 |
| DCU | 0.00087 | 0.0017 | 0.0020 | 0.0026 | 0.0033 | 0.0052 | 0.0035 |
| DMAC | 0.0033 | 0.0038 | 0.0039 | 0.0040 | 0.0042 | 0.0044 | 0.0042 |
| DACE | 0.00068 | 0.0011 | 0.0013 | 0.0015 | 0.0018 | 0.0023 | 0.0019 |
| DCUP | 0.000058 | 0.00011 | 0.0011 | 0.0027 | 0.0068 | 0.0095 | 0.019 |
| DOTH | 0.00090 | 0.00090 | 0.0022 | 0.0032 | 0.0060 | 0.0078 | 0.0091 |

| Titration→  Reaction↓ | MBCS | M0 | M10 | M50 | M100 | M175 | M250 | Ave | Stdev |
| --- | --- | --- | --- | --- | --- | --- | --- | --- | --- |
| kBAMAC | 0.0051 | 0.0051 | 0.0051 | 0.0051 | 0.0051 | 0.0051 | 0.0051 | 0.0051 | 0.00000034 |
| kBAACE | 0.0062 | 0.0061 | 0.0062 | 0.0062 | 0.0062 | 0.0062 | 0.0062 | 0.0062 | 0.0000015 |
| kBAOTH | 0.0090 | 0.0045 | 0.0074 | 0.0069 | 0.0095 | 0.0097 | 0.010 | 0.0082 | 0.00055 |
| kBACUP | 0.0011 | 0.00063 | 0.0038 | 0.0047 | 0.0075 | 0.0064 | 0.014 | 0.0055 | 0.0066 |
| kBCTR | 0.0014 | 0.0010 | 0.00097 | 0.00085 | 0.00075 | 0.00059 | 0.00073 | 0.00097 | 0.00000080 |
| kCUIN1 | 0.038 | 0.032 | 0.038 | 0.038 | 0.038 | 0.038 | 0.038 | 0.037 | 0.0000096 |
| kCUIN2 | 0 | 0.0010 | 0.0019 | 0.029 | 0.079 | 0.11 | 0.18 | 0.066 | 0.091 |
| kMMACF | 0.39 | 0.38 | 0.39 | 0.39 | 0.40 | 0.39 | 0.39 | 0.39 | 0.0023 |
| kMACR | 0.39 | 0.39 | 0.39 | 0.39 | 0.39 | 0.39 | 0.39 | 0.39 | 0.000062 |
| kMACEF | 0.13 | 0.13 | 0.13 | 0.13 | 0.13 | 0.13 | 0.13 | 0.13 | 0.000046 |
| kMACER | 0.13 | 0.13 | 0.13 | 0.13 | 0.13 | 0.13 | 0.13 | 0.13 | 0.000089 |
| kMCUPF | 0.074 | 0.080 | 0.12 | 0.18 | 0.27 | 0.37 | 1.3 | 0.34 | 0.61 |
| kMCUPR | 0.11 | 0.11 | 0.11 | 0.12 | 0.12 | 0.11 | 0.12 | 0.11 | 0.0016 |
| kMOTHF | 0.13 | 0.15 | 0.22 | 0.35 | 0.56 | 0.80 | 2.6 | 0.70 | 1.3 |
| kMOTHR | 0.22 | 0.22 | 0.22 | 0.22 | 0.22 | 0.22 | 0.22 | 0.22 | 0.000000012 |
|  |  |  |  |  |  |  |  |  |  |
| Simulated  with logistical functions included |  |  |  |  |  |  |  |  |  |
| kBACUP | 1.9E-08 | 1.7E-06 | 7.2E-06 | 0.00022 | 0.0072 | 0.028 | 0.014 |  |  |
| kCUIN2 | 1.0E-08 | 2.5E-06 | 1.5E-05 | 0.00096 | 0.077 | 0.36 | 0.18 |  |  |
| kMCUPF | 1.2E-12 | 9.3E-09 | 1.7E-07 | 0.00016 | 0.27 | 2.6 | 1.3 |  |  |
| kMOTHF | 0.0020 | 0.024 | 0.053 | 0.33 | 1.9 | 5.3 | 2.7 |  |  |

**Table S7: Rate-Constants** (μM/min)

**Table S8: Kinetic Parameters used for the dynamical system**

| Parameter | Value | Units |
| --- | --- | --- |
| kBAMAC | 0.0051 | μM/min |
| kBAACE | 0.0062 | μM/min |
| kBAOTH | 0.0082 | μM/min |
| kBACUP_in_ | 0.012 | μM/min |
| kBCTR | 0.00097 | μM/min |
| kCUIN1 | 0.037 | μM/min |
| kCUIN2_in_ | 0.15 | μM/min |
| kMMACF | 0.39 | μM/min |
| kMMACR | 0.39 | μM/min |
| kMACEF | 0.13 | μM/min |
| kMACER | 0.13 | μM/min |
| kMCUPF_in_ | 2.0 | μM/min |
| kMCUPR | 0.11 | μM/min |
| kMOTHF_in_ | 2.0 | μM/min |
| kMOTHR | 0.23 | μM/min |
| K_M1_ | 2 | μM |
| K_M2_ | 35 | μM |
| α_cell_ | 0.0033 | min^-1^ |
| [aACE]_m_ | 1.5 | μM |
| [ACE]_m_ | 0.38 | μM |
| [aCUP]_m_ | 0.80 | μM |
| [CUP]_m_ | 0.34 | μM |
| [aOTH]_m_ | 1.6 | μM |
| [OTH]_m_ | 0.67 | μM |
| [aMAC]_m_ | 0.37 | μM |
| [MAC]_m_ | 1.2 | μM |
| [CTR]_m_ | 0.29 | μM |
| [CU]_m_ | 0.59 | μM |
| Sen_sp_ (kBACUP) | 0.86 | μM |
| Sen_sp_ (kCUIN2) | 99 | μM |
| Sen_sp_ (kMCUPF) | 1.4 | μM |
| Sen_sp_ (kMOTHF) | 1.1 | μM |
| *n* (kBACUP) | 4.6 | μM^-1^ |
| *n* (kCUIN2) | 0.037 | μM^-1^ |
| *n* (kMCUPF) | 4.0 | μM^-1^ |
| *n* (kMOTHF) | 6.3 | μM^-1^ |
